# Heuristic recurrent algorithms for photonic Ising machines

**DOI:** 10.1038/s41467-019-14096-z

**Published:** 2020-01-14

**Authors:** Charles Roques-Carmes, Yichen Shen, Cristian Zanoci, Mihika Prabhu, Fadi Atieh, Li Jing, Tena Dubček, Chenkai Mao, Miles R. Johnson, Vladimir Čeperić, John D. Joannopoulos, Dirk Englund, Marin Soljačić

**Affiliations:** 1 Research Laboratory of Electronics, Massachusetts Institute of Technology, 50 Vassar Street, Cambridge, MA 02139 USA; 2 Department of Electrical Engineering and Computer Science, Massachusetts Institute of Technology, 77 Massachusetts Avenue, Cambridge, MA 02139 USA; 3 Department of Physics, Massachusetts Institute of Technology, 77 Massachusetts Avenue, Cambridge, MA 02139 USA; 4 Department of Mathematics, Massachusetts Institute of Technology, 77 Massachusetts Avenue, Cambridge, MA 02139 USA; 5 Institute for Soldier Nanotechnologies, 500 Technology Square, Cambridge, MA 02139 USA

**Keywords:** Photonic devices, Statistical physics, thermodynamics and nonlinear dynamics

## Abstract

The inability of conventional electronic architectures to efficiently solve large combinatorial problems motivates the development of novel computational hardware. There has been much effort toward developing application-specific hardware across many different fields of engineering, such as integrated circuits, memristors, and photonics. However, unleashing the potential of such architectures requires the development of algorithms which optimally exploit their fundamental properties. Here, we present the Photonic Recurrent Ising Sampler (PRIS), a heuristic method tailored for parallel architectures allowing fast and efficient sampling from distributions of arbitrary Ising problems. Since the PRIS relies on vector-to-fixed matrix multiplications, we suggest the implementation of the PRIS in photonic parallel networks, which realize these operations at an unprecedented speed. The PRIS provides sample solutions to the ground state of Ising models, by converging in probability to their associated Gibbs distribution. The PRIS also relies on intrinsic dynamic noise and eigenvalue dropout to find ground states more efficiently. Our work suggests speedups in heuristic methods via photonic implementations of the PRIS.

## Abstract (web summary)

Application-specific computational hardware helps to solve the limitations of conventional electronics in solving difficult calculation problems. Here the authors present a general heuristic algorithm to solve NP-Hard Ising problems in a photonics implementation.

## Introduction

Heuristic methods—probabilistic algorithms with stochastic components—are a cornerstone of both numerical methods in statistical physics^1^ and NP-Hard optimization^2^. Broad classes of problems in statistical physics, such as growth patterns in clusters^3^, percolation^4^, heterogeneity in lipid membranes^5^, and complex networks^6^, can be described by heuristic methods. These methods have proven instrumental for predicting phase transitions and the critical exponents of various universality classes – families of physical systems exhibiting similar scaling properties near their critical temperature^1^. These heuristic algorithms have become popular, as they typically outperform exact algorithms at solving real-world problems^7^. Heuristic methods are usually tailored for conventional electronic hardware; however, a number of optical machines have recently been shown to solve the well-known Ising^8,9^ and Traveling Salesman problems^10,11^. For computationally demanding problems, these methods can benefit from parallelization speedups^1,12^, but the determination of an efficient parallelization approach is highly problem-specific^1^.

Half a century before the contemporary Machine Learning Renaissance^13^, the Little^14^ and then the Hopfield^15,16^ networks were considered as early architectures of recurrent neural networks (RNN). The latter was suggested as an algorithm to solve combinatorially hard problems, as it was shown to deterministically converge to local minima of arbitrary quadratic Hamiltonians of the form

1 $${H}^{(K)}=-\frac{1}{2}\sum _{1\le i,j\le N}{\sigma }_{i}{K}_{ij}{\sigma }_{j},$$

which is the most general form of an Ising Hamiltonian in the absence of an external magnetic field^17^. In Eq. (1), we equivalently denote the set of spins as *σ* ∈ {−1, 1}^*N*^ or *S* ∈ {0, 1}^*N*^ (with *σ* = 2*S*−1), and *K* is a *N* × *N* real symmetric matrix.

In the context of physics, Ising models describe the interaction of many particles in terms of the coupling matrix *K*. These systems are observed in a particular spin configuration *σ* with a probability given by the Gibbs distribution $p(\sigma )\propto \exp (-\beta {H}^{(K)}(\sigma ))$, where *β* *=* 1/(*k*_*B*_*T*), with *k*_*B*_ the Boltzmann constant and *T* the temperature. At low temperature, when *β* → *∞*, the Gibbs probability of observing the system in its ground state approaches 1, thus naturally minimizing the quadratic function in Eq. (1). As similar optimization problems are often encountered in computer science^2,7^, a natural idea is to engineer physical systems with dynamics governed by an equivalent Hamiltonian. Then, by sampling the physical system, one can generate candidate solutions to the optimization problem. This analogy between statistical physics and computer science has nurtured a great variety of concepts in both fields^18^, for instance, the analogy between neural networks and spin glasses^15,19^.

Many complex systems can be formulated using the Ising model^20^—such as ferromagnets^17,21^, liquid-vapor transitions^22^, lipid membranes^5^, brain functions^23^, random photonics^24^, and strongly-interacting systems in quantum chromodynamics^25^. From the perspective of optimization, finding the spin distribution minimizing *H*^(*K*)^ for an arbitrary matrix *K* belongs to the class of NP-hard problems^26^.

Hopfield networks deterministically converge to a local minimum, thus making it impossible to scale such networks to deterministically find the global minimum^27^—thus jeopardizing any electronic^16^ or optical^28^ implementation of these algorithms. As a result, these early RNN architectures were soon superseded by heuristic (such as Metropolis-Hastings (MH)) and metaheuristic methods (such as simulated annealing (SA)^29^, parallel tempering^30^, genetic algorithms^31^, Tabu search^32^ and local-search-based algorithms^33^), usually tailored for conventional electronic hardware. Even still, heuristic methods struggle to solve large problems, and could benefit from nanophotonic hardware demonstrating parallel, low-energy, and high-speed computations^34–36^.

Here, we propose a photonic implementation of a passive RNN, which models the arbitrary Ising-type Hamiltonian in Eq. (1). We propose a fast and efficient heuristic method for photonic analog computing platforms, relying essentially on iterative matrix multiplications. Our heuristic approach also takes advantage of optical passivity and dynamic noise to find ground states of arbitrary Ising problems and probe their critical behaviors, yielding accurate predictions of critical exponents of the universality classes of conventional Ising models. Our algorithm presents attractive scaling properties when benchmarked against conventional algorithms, such as MH. Our findings suggest a novel approach to heuristic methods for efficient optimization and sampling by leveraging the potential of matrix-to-vector accelerators, such as parallel photonic networks^34^. We also hint at a broader class of (meta)heuristic algorithms derived from the PRIS, such as combined simulated annealing on the noise and eigenvalue dropout levels. Our algorithm can also be implemented in a competitive manner on fast parallel electronic hardware, such as FPGAs and ASICs.

## Results

### Photonic computational architecture

**Fig. 1:**
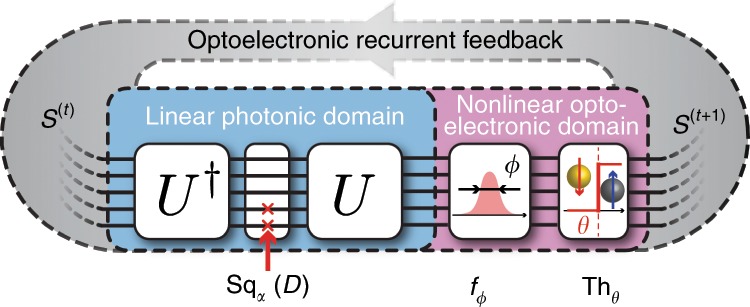
Operation principle of the PRIS. A photonic analog signal, encoding the current spin state *S*^(*t*)^, goes through transformations in linear photonic and nonlinear optoelectronic domains. The result of this transformation *S*^(*t*+1)^ is recurrently fed back to the input of this passive photonic system.

The proposed architecture of our photonic network is shown in Fig. 1. This photonic network can map arbitrary Ising Hamiltonians described by Eq. (1), with *K*_*ii*_  = 0 (as diagonal terms only contribute to a global offset of the Hamiltonian, see Supplementary Note 1). In the following, we will refer to the eigenvalue decomposition of *K* as *K*  = *UDU*^†^, where *U* is a unitary matrix, *U*^†^ its transpose conjugate, and *D* a real-valued diagonal matrix. The spin state at time step *t*, encoded in the phase and amplitude of *N* parallel photonic signals *S*^(*t*)^ ∈ {0, 1}^*N*^, first goes through a linear symmetric transformation decomposed in its eigenvalue form 2*J* = *U*Sq_*α*_(*D*)*U*^†^, where Sq_*α*_(*D*) is a diagonal matrix derived from *D*, whose design will be discussed in the next paragraphs. The signal is then fed into nonlinear optoelectronic domain, where it is perturbed by a Gaussian distribution of standard deviation *ϕ* (simulating noise present in the photonic implementation) and is imparted a nonlinear threshold function Th_*θ*_ (Th_*θ*_(*x*) = 1 if *x* > *θ*, 0 otherwise). The signal is then recurrently fed back to the linear photonic domain, and the process repeats. The static unit transformation between two time steps *t* and *t* + 1 of this RNN can be summarized as

2 $${X}^{(t)} 	\sim {\mathcal{N}}(2J{S}^{(t)}| \phi ),\\ {S}^{(t+1)}	={{\rm{Th}}}_{\theta }({X}^{(t)})$$

where ${\mathcal{N}}(x| \phi )$ denotes a Gaussian distribution of mean *x* and standard deviation *ϕ*. We call this algorithm, which is tailored for a photonic implementation, the Photonic Recurrent Ising Sampler (PRIS). The detailed choice of algorithm parameters is described in the Supplementary Note 2.

This simple recurrent loop can be readily implemented in the photonic domain. For example, the linear photonic interference unit can be realized with MZI networks^34,37–39^, diffractive optics^40,41^, ring resonator filter banks^42–44^, and free space lens-SLM-lens systems^45,46^; the diagonal matrix multiplication Sq_*α*_(*D*) can be implemented with an electro-optical absorber, a modulator or a single MZI^34,47,48^; the nonlinear optoelectronic unit can be implemented with an optical nonlinearity^47–51^, or analog/digital electronics^52–55^, for instance by converting the optical output to an analog electronic signal, and using this electronic signal to modulate the input^56^. The implementation of the PRIS on several photonic architectures and the influence of heterogeneities, phase bit precision, and signal to noise ratio on scaling properties are discussed in the Supplementary Note 5. In the following, we will describe the properties of an ideal PRIS and how design imperfections may affect its performance.

### General theory of the PRIS dynamics

The long-time dynamics of the PRIS is described by an effective Hamiltonian *H*_*L*_ (see refs. ^19,58^ and Supplementary Note 2). This effective Hamiltonian can be computed by performing the following steps. First, calculate the transition probability of a single spin from Eq. (2). Then, the transition probability from an initial spin state *S*^(*t*)^ to the next step *S*^(*t*+1)^ can be written as

3 $${W}^{(0)}\left({S}^{(t+1)}| {S}^{(t)}\right)=\frac{{e}^{-\beta {H}^{0}\left({S}^{(t+1)}| {S}^{(t)}\right)}}{\sum _{S}{e}^{-\beta {H}^{0}\left(S| {S}^{(t)}\right)}},$$

4 $${H}^{0}\left(S| S^{\prime} \right)=-\sum _{1\le i,j\le N}{\sigma }_{i}\left(S\right){J}_{ij}{\sigma }_{j}\left(S^{\prime} \right),$$

where $S,S^{\prime}$ denote arbitrary spin configurations. Let us emphasize that, unlike *H*^(*K*)^(*S*), the transition Hamiltonian ${H}^{(0)}\left(S| S^{\prime} \right)$ is a function of two spin distributions *S* and $S^{\prime}$. Here, *β* = 1∕(*kϕ*) is analogous to the inverse temperature from statistical mechanics, where *k* is a constant, only depending on the noise distribution (see Supplementary Table 1). To obtain Eqs. (3), (4), we approximated the single spin transition probability by a rescaled sigmoid function and have enforced the condition *θ*_*i*_ = ∑_*j*_*J*_*ij*_. In the Supplementary Note 2, we investigate the more general case of arbitrary threshold vectors *θ*_*i*_ and discuss the influence of the noise distribution.

One can easily verify that this transition probability obeys the triangular condition (or detailed balance condition) if *J* is symmetric *J*_*ij*_ = *J*_*ji*_. From there, an effective Hamiltonian *H*_*L*_ can be deduced following the procedure described by Peretto^58^ for distributions verifying the detailed balance condition. The effective Hamiltonian *H*_*L*_ can be expanded, in the large noise approximation (*ϕ* ≫ 1, *β* ≪ 1), into *H*_2_:

5 $${H}_{L}=-\frac{1}{\beta }\sum _{i}\mathrm{log}\cosh \left(\beta \sum _{j}{J}_{ij}{\sigma }_{j}\right),$$

6 $${H}_{2}=-\frac{\beta }{2}\sum _{1\le i,j\le N}{\sigma }_{i}{[{J}^{2}]}_{ij}{\sigma }_{j}.$$

Examining Eq. (6), we can deduce a mapping of the PRIS to the general Ising model shown in Eq. (1) since ${H}_{2}=\beta {H}^{({J}^{2})}$. We set the PRIS matrix *J* to be a modified square-root of the Ising matrix *K* by imposing the following condition on the PRIS

7 $${{\rm{Sq}}}_{\alpha }(D)=2{\rm{Re}}\, (\sqrt{D+\alpha \Delta }).$$

We add a diagonal offset term *αΔ* to the eigenvalue matrix *D*, in order to parametrize the number of eigenvalues remaining after taking the real part of the square root. Since lower eigenvalues tend to increase the energy, they can be dropped out so that the algorithm spans the eigenspace associated with higher eigenvalues. We chose to parametrize this offset as follows: $\alpha \in {\mathbb{R}}$ is called the eigenvalue dropout level, a hyperparameter to select the number of eigenvalues remaining from the original coupling matrix *K*, and *Δ* > 0 is a diagonal offset matrix. For instance, *Δ* can be defined as the sum of the off-diagonal terms of the Ising coupling matrix *Δ*_*ii*_ = Σ_*j*≠*i*_∣*K*_*ij*_∣. The addition of *Δ* only results in a global offset on the Hamiltonian. The purpose of the *Δ* offset is to make the matrix in the square root diagonally dominant, thus symmetric positive definite, when *α* is large and positive. Thus, other definitions of the diagonal offset could be proposed. When *α* → 0, some lower eigenvalues are dropped out by taking the real part of the square root (see Supplementary Note 3); we show below that this improves the performance of the PRIS. We will specify which definition of *Δ* is used in our study when *α* ≠ 0. When choosing this definition of Sq_*α*_(*D*) and operating the PRIS in the large noise limit, we can implement any general Ising model (Eq. (1)) on the PRIS (Eq. (6)).

It has been noted that by setting Sq_*α*_(*D*) = *D* (i.e., the linear photonic domain matrix amounts to the Ising coupling matrix 2*J* = *K*), the free energy of the system equals the Ising free energy at any finite temperature (up to a factor of 2, thus exhibiting the same ground states) in the particular case of associative memory couplings^19^ with finite number of patterns and in the thermodynamic limit, thus drastically constraining the number of degrees of freedom on the couplings. This regime of operation is a direct modification of the Hopfield network, an energy-based model where the couplings between neurons is equal to the Ising coupling between spins. The essential difference between the PRIS in the configuration Sq_*α*_(*D*) = *D* and a Hopfield network is that the former relies on synchronous spin updates (all spins are updated at every step, in this so-called Little network^14^) while the latter relies on sequential spin updates (a single randomly picked spin is updated at every step). The former is better suited for a photonic implementation with parallel photonic networks.

In this regime of operation, the PRIS can also benefit from computational speed-ups, if implemented on a conventional architecture, for instance if the coupling matrix is sparse. However, as has been pointed out in theory^19^ and by our simulations (see Supplementary Note 4, Supplementary Fig. 7), some additional considerations should be taken into account in order to eliminate non-ergodic behaviors in this system. As the regime of operation described by Eq. (7) is general to any coupling, we will use it in the following demonstrations.

### Finding the ground state of Ising models with the PRIS

**Fig. 2:**
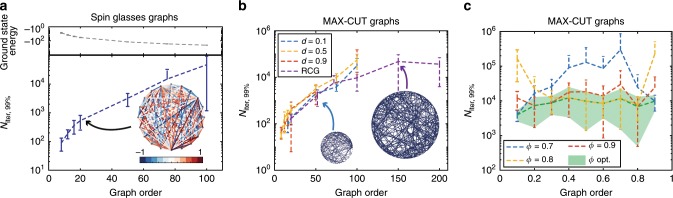
Scaling performance of the PRIS. (**a**, top) Ground state energy versus graph order of random spin glasses. A sample graph is shown as an inset in (**a**, bottom): a fully-connected spin glass with uniformly-distributed continuous couplings in [−1, 1]. N_iter, 99%_ versus graph size for spin glasses (**a**, bottom) and MAX-CUT graphs (**b**). **c** N_iter, 99%_ versus graph density for MAX-CUT graphs and *N* = 75. The graph density is defined as *d* = 2∣*E*∣∕(*N*(*N* − 1)), ∣*E*∣ being the number of undirected edges. RCG denotes Random Cubic Graphs, for which ∣*E*∣ = 3*N*∕2. Ground states are determined with the exact solver BiqMac^57^ (see Methods section). In this analysis, we set *α* = 0, and for each set of density and graph order we ran 10 graph instances 1000 times. The number of iterations to find the ground state is measured for each run and N_iter, *q*_ is defined as the *q*-th quantile of the measured distribution.

We investigate the performance of the PRIS on finding the ground state of general Ising problems Eq. (1) with two types of Ising models: MAX-CUT graphs, which can be mapped to an instance of the unweighted MAX-CUT problem^9^ and all-to-all spin glasses, whose connections are uniformly distributed in [−1, 1] (an example illustration of the latter is shown as an inset in Fig. 2a). Both families of models are computationally NP-hard problems^26^, thus their computational complexity grows exponentially with the graph order *N*.

The number of steps necessary to find the ground state with 99% probability, N_iter, 99%_ is shown in Fig. 2a–b for these two types of graphs (see definition in Supplementary Note 4 and in the Methods section). As the PRIS can be implemented with high-speed parallel photonic networks, the on-chip real time of a unit step can be less than a nanosecond^34,59^ (and the initial setup time for a given Ising model is typically of the order of microseconds with thermal phase shifters^60^). In such architectures, the PRIS would thus find ground states of arbitrary Ising problems with graph orders *N* ~ 100 within less than a millisecond. We also show that the PRIS can be used as a heuristic ground state search algorithm in regimes where exact solvers typically fail (*N* ~ 1000) and benchmark its performance against MH and conventional metaheuristics (SA) (see Supplementary Note 6). Interestingly, both classical and quantum optical Ising machines have exhibited limitations in their performance related to the graph density^9,61^. We observe that the PRIS is roughly insensitive to the graph density, when optimizing the noise level *ϕ* (see Fig. 2c, shaded green area). A more comprehensive comparison should take into account the static fabrication error in integrated photonic networks^34^ (see also Supplementary Note 5), even though careful calibration of their control electronics can significantly reduce its impact on the computation^62,63^.

### Influence of the noise and eigenvalue dropout levels

**Fig. 3:**
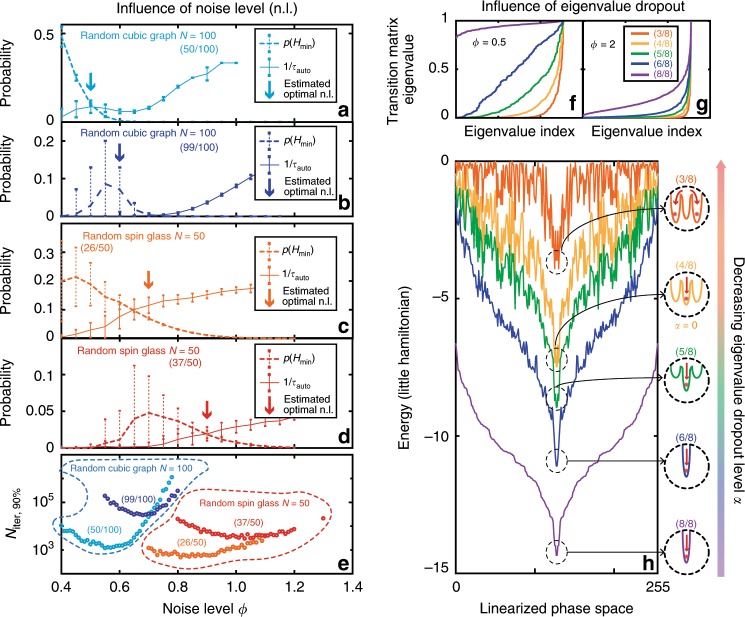
Influence of noise and eigenvalue dropout levels. **a**–**d** Probability of finding the ground state, and the inverse of the autocorrelation time as a function of noise level *ϕ* for a sample Random Cubic Graph^9^ (*N* = 00, (50/100) eigenvalues (**a**), (99/100) eigenvalues (**b**), and a sample spin glass (*N* = 50, (37/100) eigenvalues (**c**), (26/100) eigenvalues (**d**)). The arrows indicate the estimated optimal noise level, from Eq. (8), taking ${\tau }_{{\rm{eq}}}^{E}$ to be constant. For this study we averaged the results of 100 runs of the PRIS with random initial states with error bars representing  ± *σ* from the mean over the 100 runs. We assumed *Δ*_*ii*_ = ∑_*j*_*K*_*ij*_. (**e**): N_iter, 90%_ versus noise level *ϕ* for these same graphs and eigenvalue dropout levels. **f**–**g** Eigenvalues of the transition matrix of a sample spin glass (*N* = 8) at *ϕ* = 0.5 (**f**) and *ϕ*= 2 (**g**). **h** The corresponding energy is plotted for various eigenvalue dropout levels *α*, corresponding to less than *N* eigenvalues kept from the original matrix. The inset is a schematic of the relative position of the global minimum when *α* = 1 (with (8/8) eigenvalues) with respect to nearby local minima when *α* < 1. For this study we assumed *Δ*_*ii*_ = ∑_*j*_*K*_*ij*_.

For a given Ising problem, there remain two degrees of freedom in the execution of the PRIS: the noise and eigenvalue dropout levels. The noise level *ϕ* determines the level of entropy in the Gibbs distribution probed by the PRIS $p(E)\propto \exp (-\beta (E-\phi S(E)))$, where *S*(*E*) is the Boltzmann entropy associated with the energy level *E*. On the one hand, increasing *ϕ* will result in an exponential decay of the probability of finding the ground state $p({H}_{\min },\phi )$. On the other hand, too small a noise level will not satisfy the large noise approximation *H*_*L*_ ~ *H*_2_ and result in large autocorrelation times (as the spin state could get stuck in a local minimum of the Hamiltonian). Figure 3e demonstrates the existence of an optimal noise level *ϕ*, minimizing the number of iterations required to find the ground state of a given Ising problem, for various graph sizes, densities, and eigenvalue dropout levels. This optimal noise value can be approximated upon evaluation of the probability of finding the ground state $p({H}_{\min },\phi )$ and the energy autocorrelation time ${\tau }_{{\rm{auto}}}^{E}$, as the minimum of the following heuristic

8 $${N}_{{\rm{iter}},q} \sim {\tau }_{{\rm{eq}}}^{E}(\phi )+{\tau }_{{\rm{auto}}}^{E}(\phi )\frac{\mathrm{log}\, (1-q)}{\mathrm{log}(1-p({H}_{\min },\phi ))},$$

which approximates the number of iterations required to find the ground state with probability *q* (see Fig. 3a–e). In this expression, ${\tau }_{{\rm{eq}}}^{E}(\phi )$ is the energy equilibrium (or burn-in) time. As can be seen in Fig. 3e, decreasing *α* (and thus dropping more eigenvalues, with the lowest eigenvalues being dropped out first) will result in a smaller optimal noise level *ϕ*. Comparing the energy landscape for various eigenvalue dropout levels (Fig. 3h) confirms this statement: as *α* is reduced, the energy landscape is perturbed. However, for the random spin glass studied in Fig. 3f–g, the ground state remains the same down to *α* = 0. This hints at a general observation: as lower eigenvalues tend to increase the energy, the Ising ground state will in general be contained in the span of eigenvectors associated with higher eigenvalues (see discussion in the Supplementary Note 3). Nonetheless, the global picture is more complex, as the solution of this optimization problem should also enforce the constraint *σ* ∈ {−1, 1}^*N*^. We observe in our simulations that *α* = 0 yields a higher ground state probability and lower autocorrelation times than *α* > 0 for all the Ising problems we used in our benchmark. In some sparse models, the optimal value can even be *α* < 0 (see Supplementary Fig. 3 in the Supplementary Note 4). The eigenvalue dropout is thus a parameter that constrains the dimensionality of the ground state search.

The influence of eigenvalue dropout can also be understood from the perspective of the transition matrix. Figure 3f–g shows the eigenvalue distribution of the transition matrix for various noise and eigenvalue dropout levels. As the PRIS matrix eigenvalues are dropped out, the transition matrix eigenvalues become more nonuniform, as in the case of large noise (Fig. 3g). Overall, the eigenvalue dropout can be understood as a means of pushing the PRIS to operate in the large noise approximation, without perturbing the Hamiltonian in such a way that would prevent it from finding the ground state. The improved performance of the PRIS with *α* ~ 0 hints at the following interpretation: the perturbation of the energy landscape (which affects $p({H}_{\min })$) is counterbalanced by the reduction of the energy autocorrelation time induced by the eigenvalue dropout. The existence of these two degrees of freedom suggests a realm of algorithmic techniques to optimize the PRIS operation. One could suggest, for instance, setting *α* ≈ 0, and then performing an inverse simulated annealing of the eigenvalue dropout level to increase the dimensionality of the ground state search. This class of algorithms could rely on the development of high-speed, low-loss integrated modulators^59,64–66^.

### Detecting and characterizing phase transitions with the PRIS

The existence of an effective Hamiltonian describing the PRIS dynamics Eq. (6) further suggests the ability to generate samples of the associated Gibbs distribution at any finite temperature. This is particularly interesting considering the various ways in which noise can be added in integrated photonic circuits by tuning the operating temperature, laser power, photodiode regimes of operation, etc.^52,67^. This alludes to the possibility of detecting phase transitions and characterizing critical exponents of universality classes, leveraging the high speed at which photonic systems can generate uncorrelated heuristic samples of the Gibbs distribution associated with Eqs. (5), (6). In this part, we operate the PRIS in the regime where the linear photonic matrix is equal to the Ising coupling matrix (Sq_*α*_(*D*) = *D*)^19^. This allows us to speedup the computation on a CPU by leveraging symmetry and sparsity of the coupling matrix *K*. We show that the regime of operation described by Eq. (7) also probes the expected phase transition (see Supplementary Note 4).

**Fig. 4:**
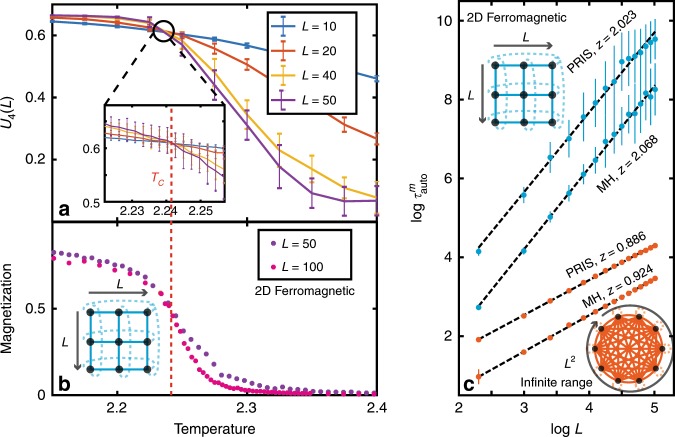
Detecting and characterizing phase transitions. **a** Binder cumulants *U*_4_(*L*) for various graph sizes L on the 2D Ferromagnetic Ising model. Their intersection determines the critical temperature of the model *T*_*C*_ (denoted by a dotted line). **b** Magnetization estimated from the PRIS for various *L*. **c** Scaling of the PRIS magnetization autocorrelation time for various Ising models, benchmarked versus the Metropolis-Hastings algorithm (MH). The complexity of a single time step scales like *N*^2^ = *L*^4^ for MH on a CPU and like *N* = *L*^2^ for the PRIS on a photonic platform. For readability, error bars in **b** are not shown (see Supplementary Note 4).

A standard way of locating the critical temperature of a system is through the use of the Binder cumulant^1^ ${U}_{4}(L)=1-\langle {m}^{4}\rangle /(3{\langle {m}^{2}\rangle }^{2})$, where $m={\sum }_{i=1}^{N}{\sigma }_{i}/N$ is the magnetization and 〈.〉 denotes the ensemble average. As shown in Fig. 4a, the Binder cumulants intersect for various graph sizes *L*^2^ = *N* at the critical temperature of *T*_*C*_ = 2.241 (compared to the theoretical value of 2.269 for the two-dimensional Ferromagnetic Ising model, i.e., within 1.3%). The heuristic samples generated by the PRIS can be used to compute physical observables of the modeled system, which exhibit the emblematic order-disorder phase transition of the two-dimensional Ising model^1,21^ (Fig. 4b). In addition, critical parameters describing the scaling of the magnetization and susceptibility at the critical temperature can be extracted from the PRIS to within 10% of the theoretical value (see Supplementary Note 4).

In Fig. 4c, we benchmark the performance of the PRIS against the well-known Metropolis-Hastings (MH) algorithm^1,68,69^. In the context of heuristic methods, one should compare the autocorrelation time of a given observable. The scaling of the magnetization autocorrelation time ${\tau }_{{\rm{auto}}}^{m}={\mathcal{O}}({L}^{z})={\mathcal{O}}({N}^{z/2})$ at the critical temperature is shown in Fig. 4c for two analytically-solvable models: the two-dimensional ferromagnetic and the infinite-range Ising models. Both algorithms yield autocorrelation time critical exponents close to the theoretical value (*z* ~ 2.1)^1^ for the two-dimensional Ising model. However, the PRIS seems to perform better on denser models such as the infinite-range Ising model, where it yields a smaller autocorrelation time critical exponent. More significantly, the advantage of the PRIS resides in its possible implementation with any matrix-to-vector accelerator, such as parallel photonic networks, so that the computational (time) complexity of a single step is ${\mathcal{O}}(N)$^34,38,39^. Thus, the computational complexity of generating an uncorrelated sample scales like ${\mathcal{O}}({N}^{1+{z}_{{\rm{PRIS}}}/2})$ for the PRIS on a parallel architecture, while it scales like ${\mathcal{O}}({N}^{2+{z}_{{\rm{MH}}}/2})$ for a sequential implementation of MH, on a CPU for instance. Implementing the PRIS on a photonic parallel architecture also ensures that the prefactor in this order of magnitude estimate is small (and only limited by the clock rate of a single recurrent step of this high-speed network). Thus, as long as *z*_PRIS_ < *z*_MH_ + 2, the PRIS exhibits a clear advantage over MH implemented on a sequential architecture.

## Discussion

To conclude, we have presented the PRIS, a photonic-based heuristic algorithm able to probe arbitrary Ising Gibbs distributions at various temperature levels. At low temperatures, the PRIS can find ground states of arbitrary Ising models with high probability. Our approach essentially relies on the use of matrix-to-vector product accelerators, such as photonic networks^34,67^, free-space optical processors^28^, FPGAs^70^, and ASICs^71^ (see comparison of time estimates in the Supplementary Note 5). We also perform a proof-of-concept experiment on a Xilinx Zynq UltraScale+ multiprocessor system-on-chip (MPSoC) ZCU104, an electronic board containing a parallel programmable logic unit (FPGA-Field Programmable Gate Arrays). We run the PRIS on large random spin glasses *N* = 100 and achieve algorithm time steps of 63 ns. This brings us closer to photonic clocks  ≲1 ns, thus demonstrating that (1) the PRIS can leverage parallel architectures of various natures, electronics and photonics; (2) the potential of hybrid parallel opto-electronic implementations. Details of the FPGA implementation and numerical experiments are given in Supplementary Note 7.

Moreover, our system requires some amount of noise to perform better, which is an unusual behavior only observed in very few physical systems. For instance, neuroscientists have conjectured that this could be a feature of the brain and spiking neural networks^72,73^. The PRIS also performs a static transformation (and the state evolves to find the ground state). This kind of computation can rely on a fundamental property of photonics—passivity—and thus reach even higher efficiencies. Non-volatile phase-change materials integrated in silicon photonic networks could be leveraged to implement the PRIS with minimal energy costs^74^.

We also suggested a broader family of photonic metaheuristic algorithms which could achieve even better performance on larger graphs (see Supplementary Note 6). For instance, one could simulate annealing with photonics by reducing the system noise level (this could be achieved by leveraging quantum photodetection noise^67^, see discussion in Supplementary Notes 5 and 6). We believe that this class of algorithms that can be implemented on photonic networks is broader than the metaheuristics derived from MH, since one could also simulate annealing on the eigenvalue dropout level *α*.

The ability of the PRIS to detect phase transitions and probe critical exponents is particularly promising for the study of universality classes, as numerical simulations suffer from critical slowing down: the autocorrelation time grows exponentially at the critical point, thus making most samples too correlated to yield accurate estimates of physical observables. Our study suggests that this fundamental issue could be bypassed with the PRIS, which can generate a very large number of samples per unit time—only limited by the bandwidth of active silicon photonics components.

The experimental realization of the PRIS on a photonic platform would require additional work compared to the demonstration of deep learning with nanophotonic circuits^34^. The noise level can be dynamically induced by several well-known sources of noise in photonic and electronic systems^52^. However, attaining a low enough noise due to heterogeneities in a static architecture, and characterizing the noise level are two experimental challenges. Moreover, the PRIS requires an additional homodyne detection unit, in order to detect both the amplitude and the phase of the output signal from the linear photonic domain. Nonetheless, these experimental challenges do not impact the promising scaling properties of the PRIS, since various photonic architectures have recently been proposed^34,40,45,67,75^, giving a new momentum to photonic computing.

## Methods

### Numerical simulations

To evaluate the performance of the algorithm on several Ising problems, we simulate the execution of an ideal photonic system, performing computations without static error. The noise is artificially added after the matrix multiplication unit and follows a Gaussian distribution, as discussed above. This results in an algorithm similar to the one described in the section II of this work.

In the main text, we present the scaling performance of the PRIS as a function of the graph order. For each graph order and density, we generate 10 random samples with these properties. We then optimize the noise level (minimizing N_iter, 99%_) on a random sample graph and generate a total of 10 samples for each pair of graph order/density. The optimal value of *ϕ* is shown in Supplementary Fig. 2 in Supplementary Note 4.

For each randomly generated graph, we first compute its ground state with the online platform BiqMac^57^. We then make 100 measurements of the number of steps required (with a random initial state) to get to this ground state. From these 1000 runs, we define the estimate of finding the ground state of the problem with *q* percent probability N_iter, *q*_ as the *q*-th quantile.

Also in the main text, we study the influence of eigenvalue dropout and of the noise level on the PRIS performance. We show that the optimal level of eigenvalue dropout is usually *α* < 1, and around *α* = 0. In some cases, it can even be *α* *<* 0 as we show in Supplementary Fig. 3 in Supplementary Note 4 where the optimal (*α*, *ϕ*) = (−0.15, 0.55) for a random cubic graph with *N* = 52. In addition to Fig. 3f–h from the main text showing the influence of eigenvalue dropout on a random spin glass, the influence of dropout on a random cubic graph is shown in Supplementary Fig. 4 in Supplementary Note 4. Similar observations can be made, but random cubic graphs, which show highly degenerated hamiltonian landscapes, are more robust to eigenvalue dropout. Even with *α* *=* −0.8, in the case shown in Supplementary Fig. 4 in Supplementary Note 4 the ground state remains unaffected.

### Others

Further details on generalization of the theory of the PRIS dynamics, construction of the weight matrix *J*, numerical simulations, scaling performance of the PRIS, and comparison of the PRIS to other (meta)heuristics algorithms can be found in the Supplementary Notes 1–7.

## Supplementary information

Supplementary Information

## Data Availability

The data that support the plots within this paper and other findings of this study are available from the corresponding authors upon reasonable request.

## Code availability

The code that supports the plots within this paper and other findings of this study are available from the corresponding authors upon reasonable request.
