# Supplementary material for: Heuristic recurrent algorithms for photonic Ising machines
Source: Nat Commun. 2020 Jan 14;11:249. doi: 10.1038/s41467-019-14096-z (PMC6959305; doi:10.1038/s41467-019-14096-z)
Supplement: Supplementary file 1 — Supplementary Information [file 41467_2019_14096_MOESM1_ESM.pdf]

Supplementary Information for:
Heuristic Recurrent Algorithms for Photonic Ising Machines

by Roques-Carmes et al.

CONTENTS

Supplementary Note 1: Theoretical preliminaries 3

Supplementary Note 2: General theory of Photonic Recurrent Ising Sampler (PRIS) 4

Supplementary Note 3: Construction of the weight matrix 11

Supplementary Note 4: Numerical simulations 14

Supplementary Note 5: Scaling of the PRIS performance on several photonic architectures 20

Supplementary Note 6: Comparison of the PRIS to several (meta)heuristics 23

Supplementary Note 7: Implementation of the PRIS on FPGA 25

Supplementary References 30

SUPPLEMENTARY NOTE 1: THEORETICAL PRELIMINARIES

Definition of an Ising problem

The general definition of an Ising problem is the following: given a matrix $(K_{ij})_{(i,j)\in I^2}$, and a vector $\{b_i\}_{i\in I}$ where I is a finite set with cardinality $|I| = N$, we want to find the spin distribution $\{\sigma_i\}_{i\in I} \in \{-1,1\}^N$ minimizing the following Hamiltonian function:

$$H_{K,b}(\{\sigma_i\}) = -\sum_{1\leq i<j\leq N} \sigma_i K_{ij} \sigma_j - \sum_i b_i \sigma_i \tag{1}$$

$$= -\frac{1}{2} \sum_{1\leq i,j\leq N} \sigma_i K_{ij} \sigma_j - \sum_i b_i \sigma_i. \tag{2}$$

In statistical mechanics, this model represents the energy of an interacting set of spins. The coupling term K_{ij} represents the coupling between spins i and j. In the general definition of the Ising model, the coupling matrix K is assumed to be symmetric. We could also assume that it has a null diagonal, as it would only add a constant offset to the Hamiltonian. We are not making this extra assumption, as it will prove later to be useful. An external magnetic field b_i can be applied, which breaks the symmetry of the problem. This general class of Ising problems is NP-hard[1] and many subclasses of this problem exhibit a similar complexity. In this work, we will characterize our optical algorithms on two subclasses of problems:

- ▷ General antiferromagnets, for which the coupling K can only take two discrete values $K_{ij} \in \{0,-1\}$, and $b_i = 0$ for all i. As every problem in this subclass is equivalent to an unweighted MAX-CUT problem, and that MAX-CUT is known to be NP-hard[1], this subclass is already NP-hard.
- ▷ Spin glasses, for which the coupling K can take on continuous values in the range $[-1,1]$, and $b_i = 0$.

However, our approach can be easily extended to any coupling matrix K. The possible extension to non-zero external magnetic fields will also be discussed. A complete study of the hardness of the various subclasses of Ising problems can be found in Ref.[2].

Mapping to the MAX-CUT problem

The MAX-CUT problem of a weighted undirected graph can be phrased in terms of its adjacency matrix A:

$$\text{Find } \operatorname{argmax}_\sigma \frac{1}{4} \sum_{ij} A_{ij}(1-\sigma_i\sigma_j) := \operatorname{argmax}_\sigma C_A(\sigma), \tag{3}$$

where $\sigma \in \{-1,1\}^N$ is a spin vector. The value of this MAX-CUT problem is called $C_{\max}$. More intuitively, this problem can be interpreted as finding a subset of vertices of the graph, such that the number of edges connecting this subset to its complementary is maximized. The vertices of this subset (resp. of its complementary set) will have spin up (resp. spin down).

We can map the general Ising problem (spin glass) (Equation (2)) to any weighted MAX-CUT problem. This mapping is useful because the publicly-available solver we use to find the Ising ground state works in terms of the MAX-CUT problem[3]. The energy of the Ising ground state and the MAX-CUT are related as follows:

$$H_{\min} = -\frac{1}{2} \sum_{ij} K_{ij} - 2C_{\max}, \tag{4}$$

where $K = -A$. From this linear relation, we also deduce that the solution of the weighted MAX-CUT problem and of the general Ising model (spin glass) are the same:

$$\operatorname{argmax}_\sigma C_A(\sigma) = \operatorname{argmin}_\sigma H^{(K)}(\sigma). \tag{5}$$

SUPPLEMENTARY NOTE 2: GENERAL THEORY OF PHOTONIC RECURRENT ISING SAMPLER (PRIS)

Optical Parallel Recurrent MCMC algorithm

The Little and Hopfield networks[4,5] are two early forms of recurrent neural networks, originally designed to understand and model associative memory processes. In its general acceptance, the Little network can be understood as a synchronous version of the Hopfield network. Using the latter to solve the NP-hard Ising problem was previously investigated[6,7]. The behavior of both networks was later studied from a statistical mechanical perspective by P. Peretto[8] and D. J. Amit and colleagues[9]. We here generalize their study to the case of noisy, synchronous Hopfield network, or noisy Little network[8] and investigate the possibility of sampling hard Ising problems with a parallel, recurrent optical system. In the following, we equivalently use the term "neuron" and "spin" in the framework of spin glass models of neural networks.

Our machine is applying the following algorithm:

▷ Initialize assembly of neurons $\{\sigma_i\}$ with random values. The signal is coded into optical domain with the reduced spins $S_i = (\sigma_i + 1)/2 \in \{0, 1\}$.

▷ At each step of the algorithm, each neuron is applied a potential v_i (random variable) whose expected value is given by a matrix multiplication with $C = 2J$:

$$\bar{v}_i = \sum_j C_{ij} S_j = \sum_{j=1}^{N} J_{ij}\sigma_j + \sum_{j=1}^{N} C_{ij}/2 \tag{6}$$

and whose probability density is given by the function f_ϕ with mean $\bar{v}_i$ and standard deviation ϕ:

$$\int f_\phi(x)\, x\, dx = \bar{v}_i, \tag{7}$$

$$\int f_\phi(x)(x - \bar{v}_i)^2\, dx = \phi^2. \tag{8}$$

▷ A non-linear threshold Th_θ is then applied to $\bar{v}_i$ to yield the neural state at the next time step:

$$\text{Th}_{\theta_i}(S_i) = \begin{cases} 0, & \text{if } v_i < \theta_i, \\ 1, & \text{otherwise.} \end{cases} \tag{9}$$

To summarize, the sequential transformation of the neuron state is:

$$S^{(t+1)} = \text{Th}_\theta(X^{(t)}), \tag{10}$$

$$X^{(t)} \sim \mathcal{N}(CS^t|\phi), \tag{11}$$

where $\mathcal{N}(x|\phi)$ is a gaussian distribution with mean x and standard deviation ϕ. The algorithm is fully determined by the following set of transformation variables: (C, f_φ, θ).

Determination of the Temperature factor

In this section, we compute the stationary distribution of the spin variable S^t for the general case with variables (C, f_φ, θ). We first determine the probability of a single spin-flip, knowing that the current spin state is J:

$$W(\sigma_i(I)|J) = G(h_i(J)\sigma_i(I)), \tag{12}$$

$$h_i(J) = -\sum_{j=1}^{N} J_{ij}\sigma_j(J) - h_i^0, \tag{13}$$

$$h_i^0 = \sum_j C_{ij}/2 - \theta_i, \tag{14}$$

Noise distribution	$G(x)$	$\frac{k}{2}$	$\epsilon_0 \equiv \max_{X\in\mathbb{R}} \lvert\epsilon_{\gamma_{\text{opt}}}(X)\rvert$
Logistic	$\frac{1}{1+\exp\left(\frac{\pi x}{\sqrt{3}\phi}\right)}$	$\frac{\sqrt{3}}{\pi}$	0
Gaussian	$\frac{1}{2}(1-\text{erf}(\frac{x}{\sqrt{2}\phi}))$	0.5877	0.0095
Cauchy*	$\frac{1}{\pi}\arctan(\frac{x}{\phi})+\frac{1}{2}$	1.16	0.0495
Laplace	$\begin{cases}\frac{1}{2}\exp(-\frac{\sqrt{2}x}{\phi}) & x\leq 0\\ 1-\frac{1}{2}\exp(\frac{\sqrt{2}x}{\phi}) & x\geq 0\end{cases}$	0.4735	0.0199
Uniform	$\begin{cases}0 & x\leq -\sqrt{3}\phi\\ \frac{x+\sqrt{3}\phi}{2\sqrt{3}\phi} & x\in[-\sqrt{3}\phi,\sqrt{3}\phi[\\ 1 & x\geq\sqrt{3}\phi\end{cases}$	0.6136	0.0561

Supplementary Table 1. **Summary of temperature factors for various noise distributions.** Each noise distribution is defined so that its standard deviation, when applicable, is equal to ϕ. (*the standard deviation of the Cauchy distribution is not defined, the linear dependence is measured as a function of the scaling parameter ϕ in this particular case.)

where we define G as a rescaled version of the noise cumulative density function:

$$F(x) = \int_{-\infty}^{x} f_\phi(u)\,du, \tag{15}$$

$$F_0(x) = F(x+\bar{v}_i), \tag{16}$$

$$G(x) = 1 - F_0(x). \tag{17}$$

In the following, we only assume that f_ϕ is symmetric and has a standard deviation ϕ. The symmetry assumption is not constraining as most noise distributions found in nature have a symmetric (and, in many cases, gaussian) distribution[10]. Our analysis can also be extended to noise distributions whose variance is infinite by parameterizing the distribution with another parameter, usually referred to as a "scaling" parameter (see Supplementary Table **1**). The case of f_ϕ being a gaussian distribution is discussed in Ref.[8], where the function G can be approximated by a sigmoid function when rescaling it by the adequate factor. In the general case, in order to minimize this approximation error, we choose the following rescaling factor

$$G_\gamma(x) = G(\gamma x),$$

$$1 - s(x) = \frac{1}{1+e^{x}},$$

$$\gamma_{\text{opt}} = \text{argmin}_{\gamma'} \max_{X\in\mathbb{R}} \lvert G_{\gamma'}(X) - (1-s(X))\rvert = \text{argmin}_{\gamma'} \max_{X\in\mathbb{R}} \lvert\epsilon_\gamma(X)\rvert,$$

where $s(x) = 1/(1+e^{-x})$ is the sigmoid function.

Naturally, the optimal scaling factor will depend on the standard deviation ϕ of the original probability distribution f. We numerically observe that this dependence is linear

$$\gamma_{\text{opt}} = \frac{k}{2}\,\phi. \tag{18}$$

Optimal scaling factors and corresponding errors are reported in Supplementary Table **1**. Identifying the transition probability to a sigmoid function is a necessary step to compute the effective Hamiltonian of the spin distribution[8].

Transition probability

By choosing adequately the temperature factor $\frac{k}{2}$, we minimize the error when approximating the transition probability by a sigmoid function (in the following, $h_i = h_i(J)$ and $\sigma_i = \sigma_i(I)$, for readability):

$$W(\sigma_i|J) = G_{\gamma_{\text{opt}}}\left(\frac{h_i\sigma_i}{\gamma_{\text{opt}}}\right) \tag{19}$$

$$= s\left(\frac{h_i\sigma_i}{\gamma_{\text{opt}}}\right) + \epsilon_{\gamma_{\text{opt}}}\left(\frac{h_i\sigma_i}{\gamma_{\text{opt}}}\right) \tag{20}$$

$$\approx s\left(\frac{h_i\sigma_i}{\gamma_{\text{opt}}}\right). \tag{21}$$

In the following, we will drop the subscript in γ_{opt} and will assume that its value is known (it can be determined numerically, given the noise distribution). The error function ϵ can be used as an expansion parameter, in order to estimate the accuracy of the approximation performed when neglecting this term (as is done in Ref.[8]).

Expansion of the transition probability in terms of ϵ

The transition probability can be derived by multiplying the probabilities of single spin-flips, these events being independent:

$$W(I|J) = \prod_i W(\sigma_i|J) \tag{22}$$

$$= \prod_i \left(s\left(\frac{h_i\sigma_i}{k\phi/2}\right) + \epsilon\left(\frac{h_i\sigma_i}{k\phi/2}\right)\right) \tag{23}$$

$$= W^0(I|J) + \sum_{k=1}^{N} W^k(I|J), \tag{24}$$

where $W^k(I|J)$ is the k-th order term in the expansion, assuming the error function ε is small ($\varepsilon \ll s$). The zero-th order term of this expansion gives us back the result from[8]:

$$W^0(I|J) = \frac{e^{-\beta H^0(I|J)}}{\sum_K e^{-\beta H^0(K|J)}}, \tag{25}$$

$$H^0(I|J) = -\sum_{ij} J_{ij}\sigma_i(I)\sigma_j(J) - \sum_i h_i^0\sigma_i(I), \tag{26}$$

$$\beta = \frac{1}{k\phi}. \tag{27}$$

Another way to write this zero-th order term[8] will prove to be useful later in our derivations:

$$W^0(I|J) = \frac{e^{-\beta H^0(I|J)}}{\prod_i 2\cosh(\beta h_i(J))}. \tag{28}$$

The general expression for the $k-$th order term can be derived:

$$W^k(I|J) = W^0(I|J) \sum_{j_1} \dots \sum_{j_k \notin \{j_1,\, \dots,\, j_{k-1}\}} \prod_l \epsilon\left(2\beta h_{j_l}\sigma_{j_l}\right)\left(1+\exp\left(2\beta h_{j_l}\sigma_{j_l}\right)\right). \tag{29}$$

The $N-$th order term scales, in the worst case scenario, like ϵ_0^N:

$$|W^N(I|J)| = \left|\prod_k \epsilon\left(2\beta h_{j_l}\sigma_{j_l}\right)\right| \leq \epsilon_0^N. \tag{30}$$

For $k < N$, analyzing the scaling of the $k-$th order term requires more assumptions. Let's look at the case $k = 1$, to verify that we can safely neglect higher-order terms in this expansion:

$$\left|\frac{W^1(I|J)}{W^0(I|J)}\right| \leq \sum_j \left|\epsilon\left(2\beta h_j\sigma_j\right)\left(1+\exp\left(2\beta h_j\sigma_j\right)\right)\right|. \tag{31}$$

This ratio scales exponentially with $\beta h_j\sigma_j/2$. However, in this case, the error function ϵ also goes to zero. In addition, increasing the value of $\beta h_j\sigma_j/2$ also increases the value of the Hamiltonian $H^0(I|J)$, and thus reduces the likelihood of the transition $W(I|J)$. **Thus, the larger the ratio $|\frac{W^1(I|J)}{W^0(I|J)}|$, the smaller the transition probability.** In the following, we will neglect all high order terms $k \geq 1$.

We do not extend our derivations to a general non-symmetric noise distribution in this work. We still suggest the following ideas to treat this extension:

- ▷ We could first treat the non-symmetric case as a perturbation of the symmetric case and thus derive the error on the effective Hamiltonian of the spin distribution.
- ▷ In this view, we suggest the parametrization of the skewness using the skew normal distribution[11], which is a natural extension of the toy-model symmetric noise distribution analyzed in Ref.[8].

Detailed balance condition

To determine the stationary distribution of the spin state, we first need to verify that the transition probability satisfies the triangular equality[8], equivalent to the detailed balance condition in the context of Markov Chains[12]:

$$W(I|K)W(K|J)W(J|I) = W(J|K)W(K|I)W(I|J). \tag{32}$$

This is equivalent to:

$$H(I|K) + H(K|J) + H(J|I) = H(J|K) + H(K|I) + H(I|J), \tag{33}$$

which is satisfied if J is symmetric (there is no condition on the linear term h_i^0 in the Hamiltonian). We can then deduce the effective Hamiltonian by decoupling the ratio of transition probabilities into the ratio of two terms depending on distributions I and J (by using Supplementary Equation (28))[8]

$$\frac{W^0(I|J)}{W^0(J|I)} = \frac{\exp(\beta \sum_i h_i^0 \sigma_i(I))}{\exp(\beta \sum_i h_i^0 \sigma_i(J))} \frac{\prod_i \cosh(\beta(\sum_j J_{ij}\sigma_j(I) + h_i^0)}{\prod_i \cosh(\beta(\sum_j J_{ij}\sigma_j(J) + h_i^0)} \tag{34}$$

$$= \frac{F(I)}{F(J)}, \tag{35}$$

and $H_L(I) = -\frac{1}{\beta}\ln F(I)$ gives the effective Hamiltonian describing the dynamics of the spin distribution:

$$H_L(I) = -\frac{1}{\beta}\sum_i \log\cosh(\beta(\sum_j J_{ij}\sigma_j(I) + h_i^0)) - \sum_i h_i^0 \sigma_i(I). \tag{36}$$

Effective Gibbs distribution

Assuming J is symmetric, the stationary distribution of the spin state is a Gibbs distribution given by the effective Hamiltonian $H_L(I)$[8,9]:

$$P(\{\sigma_i\}) \propto \exp\left(-\beta H_L(\{\sigma_i\})\right). \tag{37}$$

In the following, we define $||.||_k$ as the usual k-norm (where k is an integer).

Small noise approximation

In the small noise approximation ($\beta \gg 1$), the effective Hamiltonian can be simplified using the following Taylor expansion : $\log\cosh x \approx |x| - \log 2$:

$$H_L(\{\sigma_i\},\beta) \approx -\sum_i |\sum_j J_{ij}\sigma_j + h_i^0| + \frac{N}{\beta}\log 2 - \sum_i h_i^0\sigma_i \tag{38}$$

$$= -||J\vec{\sigma} + \vec{h}^0||_1 + \frac{N}{\beta}\log 2 - \vec{h}^0\cdot\vec{\sigma} := H_1(\{\sigma_i\},\beta). \tag{39}$$

Large noise approximation

In the large noise approximation ($\beta \ll 1$), the effective Hamiltonian can be simplified using the following Taylor expansion : $\log\cosh x \approx \log(1 + x^2/2) \approx x^2/2$,

$$H_L(\{\sigma_i\},\beta) \approx \underbrace{-\frac{\beta}{2}\sum_{ij}\tilde{K}_{ij}\sigma_i\sigma_j}_{\text{Quadratic term}} \underbrace{-\sum_i \sigma_i\left(\beta\sum_k J_{ki}h_k^0 + h_i^0\right)}_{\text{Linear term}} \tag{40}$$

$$- \underbrace{\frac{\beta}{2}\sum\left(h_j^0\right)^2}_{\text{Constant (independent of } \sigma)} \tag{41}$$

$$= -\frac{\beta}{2}||J\vec{\sigma} + \vec{h}^0||_2^2 - \vec{h}^0\cdot\vec{\sigma} := H_2(\{\sigma_i\},\beta), \tag{42}$$

with $\tilde{K} = J^2$.

Discussion on h_i^0

Identifying the Hamiltonian in Supplementary Equation (42) to the general Ising Hamiltonian in Supplementary Equation (2) requires solving the following system of equations:

$$\begin{cases} K_{ij} + \delta_{ij}\Delta_i = \beta \sum_k J_{ik} J_{kj} & (43) \\ b_i = \beta \sum_j J_{ki} h_k^0 + h_i^0 & (44) \\ J_{ij} = J_{ji}, & (45) \end{cases}$$

with unknown variables (h_i^0, J). δ_{ij} is the Kronecker symbol and Δ_i represents a degree of freedom on the diagonal of the Hamiltonian (which results in an additional constant term). Studying the set of general Ising Hamiltonians (K, b) (Supplementary Equation (2)) which can be mapped to an Ising network (J, h^0) in the large noise approximation (Supplementary Equation (42)) would require an extensive study that we do not carry in this work. However, one can notice that:

▷ In the case where the external field satisfies $J \cdot h^0 = 0$, the system has a trivial solution $h_i^0 = b_i$ and $J = \sqrt{K}$ (the conditions for which a square root of K can be found are discussed in the next section).

▷ In the more general case, one is given N "free" degrees of freedoms Δ_i in finding solutions to Supplementary Equation (43), while there are N constraints to solve in Supplementary Equation (44). Thus, it seems likely that, in non-degenerate cases, this system will have a non-trivial solution. However, one should make sure that while tuning the degrees of freedom Δ_i, the matrix J remains a real square root of the matrix K (see discussion in the next section on finding a square root). If not, this algorithm should be generalized to using complex-valued matrices J. We do not discuss this generalization of the algorithm in this work. However, considering that arbitrary (complex) unitary transformations can be implemented with our photonic architecture[13], this would be an interesting extension to this work.

In the following, for the sake of simplicity, we will assume $h_i^0 \equiv 0$. In this case, the large noise approximation Hamiltonian gives:

$$H_L(\{\sigma_i\}, \beta) \approx -\frac{\beta}{2} \sum_{ij} \tilde{K}_{ij} \sigma_i \sigma_j, \tag{46}$$

with $\tilde{K} = J^2$. Thus, the implementation of the Little network to model a general Ising Hamiltonian (Supplementary Equation (2)) strongly relies **on the possibility of finding a symmetric, real square root to the matrix K.**

Let us remind the reader of the assumptions we have made so far:

▷ The model is *synchronous*, or as stated in Little's original paper[4] "we shall suppose that the neurons are not permitted to fire at any random time but rather that they are synchronized such that they can only fire at some integral multiple of a period τ".

▷ The neurons have no memory of states older than their previous state (Markov process).

▷ The connections do not evolve in time (there is no learning involved).

▷ J is symmetric and $h_i^0 = 0$, which results in

$$\theta_i = \sum_j C_{ij}/2 = \sum_j J_{ij}, \tag{47}$$

from Supplementary Equation (14).

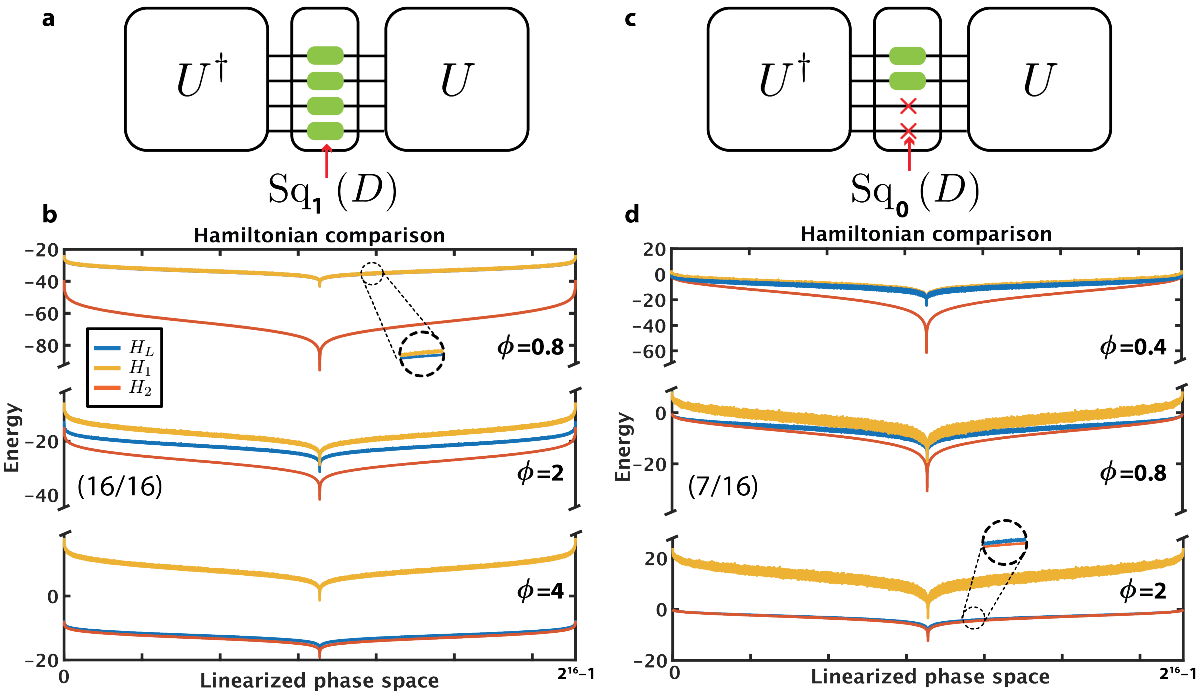

Supplementary Figure 1. **Transitioning between Hamiltonians. a:** To approximate a given Ising model in the large noise limit, one should take into account all eigenvalues. **b:** Effective Hamiltonian and its large/low-noise limit approximation when $\alpha = 1$ (with (16/16) eigenvalues). **c:** However, to reduce the noise threshold of the large noise expansion, one should drop out some eigenvalues. **d:** Effective Hamiltonian and its large/low-noise limit approximation when $\alpha = 0$ (with (7/16) eigenvalues, all negative eigenvalues being dropped out). In this Supplementary Figure, we study a random spin glass with $N = 16$ and choose $\Delta_{ii} = |\sum_j K_{ij}|$.

Inequalities between Hamiltonians

Using basic algebra and functional analysis, we can show that $\log\cosh x \leq x^2/2$ and that $\log\cosh x \geq |x| - \log 2$, which results in the following inequality on the Hamiltonians

$$H_2(\{\sigma_i\}, \beta) \leq H_L(\{\sigma_i\}, \beta) \leq H_1(\{\sigma_i\}, \beta). \tag{48}$$

By summing over spin configurations, we get the reversed inequality for the partition functions

$$Z_2(\beta) \geq Z_L(\beta) \geq Z_1(\beta). \tag{49}$$

Another inequality can be derived by using the equivalence of norms $||.||_1$ and $||.||_2$ in finite dimensions ($||.||_2 \leq ||.||_1 \leq \sqrt{N}||.||_2$):

$$0 \leq \sqrt{-\frac{2}{\beta}H_2(\{\sigma_i\}, \beta)} \leq -H_1(\{\sigma_i\}, \beta) + \frac{N}{\beta}\log 2 \leq \sqrt{\frac{-2N}{\beta}H_2(\{\sigma_i\})}. \tag{50}$$

These Hamiltonians are plotted for a sample random Ising problem in Supplementary Figure **1**.

Suggested algorithms

As suggested above, from the large noise expansion of the effective Hamiltonian, a natural way to probe the Gibbs distribution of some Ising model defined by coupling matrix K (Supplementary Equation (2) is to set the matrix of the recurrent loop C to be a modified square root of K:

$$\mathrm{Sq}_\alpha(J) = \mathrm{Re}(\sqrt{K + \alpha\Delta}), \tag{51}$$

where $\alpha \in [-1, 1]$ is the eigenvalue dropout level, a parameter we will discuss below, and Δ is a diagonal offset matrix defined as the sum of the off-diagonal term of the Ising coupling matrix $\Delta_{ii} = \sum_{j \neq i} |K_{ij}|$. An alternative solution would be to set $C = K$. This solution has been studied in the particular case of associative memory couplings[9], where it was shown that the PRIS in this configuration would be described by a free energy function equal to the Ising free energy (up to a factor of 2). We will discuss the pros and cons of both algorithms in Supplementary Note 4.

SUPPLEMENTARY NOTE 3: CONSTRUCTION OF THE WEIGHT MATRIX

We here propose a technique to build a square root of K in order to find an approximate solution to *any* Ising problem. We start with the general K Ising weight matrix K from Supplementary Equation (2). We notice that as K is symmetric and $K_{ii} = 0$, K will never obey the condition of Lemma 1.

Lemma 1 *(Diagonal dominance.) If a $A \in S_N(\mathbb{R})$ is a real symmetric matrix such that for all i, $|A_{ii}| \geq \sum_{j \neq i} |A_{ij}|$ and $A_{ii} > 0$, there exists $B \in S_N(\mathbb{R})$ such that $B^2 = A$.*

Proof 1 *Let X be a vector of size N and norm 1 such that $AX = \lambda X$ with $\lambda < 0$. We assume i_0 is the coordinate of X with maximum absolute value (and $x_{i_0} > 0$): $x_{i_0} = ||x||_\infty$. We have $\sum_j A_{i_0 j} x_j = \lambda x_{i_0}$. We thus get :*

$$\begin{aligned} ||x||_\infty |A_{i_0 i_0}| &< |A_{i_0 i_0} - \lambda| ||x||_\infty \\ &= |\sum_{j \neq i_0} A_{i_0 j} x_j| \\ &\leq \sum_{j \neq i_0} |A_{i_0 j}| ||x||_\infty. \end{aligned}$$

By simplifying this inequality, we get $|A_{i_0 i_0}| < \sum_{j \neq i_0} |A_{i_0 j}|$ which contradicts our assumption. Thus, for all X, $X^T A X \geq 0$, which means that A is positive semidefinite.

We can thus write $A = UDU^T$ where U is unitary and $D = Diag(\lambda_1, \cdots, \lambda_N)$ with $\lambda_i \geq 0$. The result is given by $B = U\sqrt{D}U^T \in S_N(\mathbb{R})$ with $\sqrt{D} = Diag(\sqrt{\lambda_1}, \cdots, \sqrt{\lambda_N})$

To be able to apply Lemma 1, we need to add diagonal terms to the Ising matrix K from Supplementary Equation 2 by defining $\tilde{K} = K + \Delta$ where Δ is a diagonal matrix such that $\tilde{K}$ verifies the assumptions of theorem 1. From there, we can define $J = \sqrt{\tilde{K}}$. In the large noise approximation, we thus get the following Hamiltonian describing the PRIS:

$$\begin{aligned} H_L(\{\sigma_i\}, \beta) \approx -\frac{\beta}{2} \sum_{ij} \tilde{K}_{ij} \sigma_i \sigma_j &= -\beta \sum_{1 \leq i < j \leq N} \tilde{K}_{ij} \sigma_i \sigma_j - \frac{\beta}{2} \sum_{1 \leq i \leq N} \tilde{K}_{ii} \sigma_i^2 \\ &= -\beta \sum_{1 \leq i < j \leq N} K \sigma_i \sigma_j - \frac{\beta}{2} \sum_{1 \leq i \leq N} \Delta_{ii}. \end{aligned}$$

Since the Δ matrix is fixed, the second term in this equation is a constant (independent of the spin distribution, given an Ising problem to minimize). The Gibbs distribution is thus $P(\{\sigma_i\}) \propto \exp(-\beta H_L(\{\sigma_i\})) \approx C_{te} \exp(-\beta H_{K,0})$ where $H_{K,0}$ is the Ising Hamiltonian defined in Supplementary Equation (2).

Discussion on the diagonal offset

The diagonal offset that is added to the original Ising matrix can be considered as a supplementary degree of freedom. To verify Lemma 1, we can choose $\Delta_{ii} = \sum_{j \neq i} |K_{ij}|$, to make sure $\tilde{K}$ is positive definite and has a real symmetric square root $J^2 = \tilde{K}$.

However, there is no reciprocal to Lemma 1. We here show that even some partial or weak reciprocals are usually wrong:

▷ The direct reciprocal of Lemma 1 is generally wrong. We consider the following counter-example:

$$A = \begin{pmatrix} 1 & -2 \\ -2 & 8 \end{pmatrix}$$

. A is symmetric, has positive diagonal elements but is not diagonally dominant (the inequality does not hold for only one diagonal element). However, A is symmetric positive definite: its determinant is 4 and its trace 9, so the sum and product of eigenvalues is positive, which means both are positive.

▷ Even a weak reciprocal of Lemma 1 is wrong:

If $A \in S_N(\mathbb{R})$, A has positive diagonal elements, such that for all i, $|A_{ii}| < \sum_{j \neq i} |A_{ij}|$, then A has at least one negative eigenvalue.

For example, one can consider :

$$A = \begin{pmatrix} 1 & 1 & 1 \\ 1 & 1 & 1 \\ 1 & 1 & 1 \end{pmatrix}$$

. A is symmetric, has positive diagonal elements, verifies: $\forall i$, $|A_{ii}| < \sum_{j \neq i} |A_{ij}|$, but still, its eigenvalues are 0, 0, and 3.

▷ One (very) weak reciprocal of Lemma 1 is the following Lemma 2. It means that any Ising matrix (that has a zero diagonal) will never have a real symmetric square root, unless we add an offset to its diagonal.

Lemma 2 *If $A \in S_N(\mathbb{R})$ (real symmetric matrix) such that for all i, $A_{ii} = 0$, then A has at least one negative (and one positive) eigenvalue.*

Proof 2 *We have $\sum_i A_{ii} = \sum_i \lambda_i = 0$ so there is at least one negative and one positive eigenvalue.*

▷ **Lemma 3 (Weak reciprocal: unweighted MAX-CUT case.)** *If K is equal to minus the adjacency matrix of a graph, then the matrix $\tilde{K} = K + \alpha\Delta$ is symmetric positive semi-definite* ***if and only if*** *$\alpha = 1$.*

Proof 3 *Let's first notice that this case still holds for a NP-hard subclass of Ising problems. In this case, $\tilde{K}$'s elements are -1 if there is a spin-spin connection and 0 otherwise. Thus, $\tilde{K} = K + \Delta$ has a zero eigenvalue: $KX = 0$ with $X = (1, 1, ..., 1)^T$ (because the sum of each row is equal to 0). We notice that for $\alpha < 1$:*

$$K + \alpha\Delta = (K + \Delta) + (\alpha - 1)\Delta. \tag{52}$$

As seen before, $K + \Delta$ has a zero eigenvalue, and $(\alpha - 1)\Delta$ only has negative eigenvalues. Using Weyl's inequalities, we can deduce that $K + \alpha\Delta$ has at least one strictly negative eigenvalue.

Studying the reciprocal of Lemma 1 gives us a better insight on what is the optimal diagonal offset Δ to add to the original Ising matrix. We can consider Δ as a supplementary degree of freedom in our algorithm. The choice of Δ suggested by this Lemma on diagonal dominance is particularly adapted for $\alpha > 0$ and Ising problems with couplings that all have the same sign. However, we notice that for large spin glasses, typically $\sum_{j \neq i} |K_{ij}|$ will be much greater than K_{ii}. Thus, $\alpha \sim -1/N$ is the lower limit for non-zero J matrices. The domain of definition of α over which the number of eigenvalues actually varies is then asymmetric. For this reason, we typically choose $\Delta_{ii} = |\sum_{j \neq i} K_{ij}|$ for large spin glasses. In this study, we will specify which definition of Δ is chosen for each Supplementary Figure and study.

Tuning the search dimensionality with eigenvalue dropout

Tuning the eigenvalue dropout gives us a way of tuning the search dimensionality. If $K = UDU^\dagger$ where U is real unitary and $D = \text{Diag}(\lambda_1, ...\lambda_N)$, we can parametrize the phase space in terms of the eigenvectors of K, which we denote as e^j (associated to eigenvalue λ_j):

$$\vec{\sigma} = \sum_j \mu_j e^j, \tag{53}$$

$$H(\vec{\sigma}) = -\frac{1}{2}\sigma^T K \sigma \tag{54}$$

$$= -\frac{1}{2}\sum_j \mu_j^2 \lambda_j. \tag{55}$$

We can rephrase the optimization problem as follows:

$$\text{Find} \quad \begin{cases} \text{argmin}_\mu - \frac{1}{2}\sum_j \mu_j^2 \lambda_j \\ \sum_j \mu_j e_i^j = \pm 1 \end{cases} \tag{56}$$

As only the positive eigenvalues can decrease the energy, we would like to conclude that only the eigenvectors associated with positive eigenvalues will contribute to minimizing the energy. However, one should make sure that the hard constraint in Supplementary Equation (56) remains satisfied. We can only conclude on the heuristic result that the ground state of the optimization probably will prefer having components in eigenspaces associated with positive eigenvalues. As reducing the eigenvalue dropout level results in dropping out the negative eigenvalues, this explains why we usually observe a better performance for a certain level around $\alpha = 0$.

Modified algorithm with tunable offset

We define Δ^0 as the minimum offset to verify the assumption of Lemma 1 and make sure the modified Ising matrix is symmetric positive semi-definite. Δ^0 is defined as:

$$\Delta^0_{ii} = \sum_{j \neq i} |A_{ij}|,$$
$$\Delta^0_{ij} = 0 \quad \text{for } i \neq j.$$

We define the modified algorithm as follows:

- ▷ $K = \tilde{K} + \alpha \Delta^0$ where $\alpha \in [0, 1]$
- ▷ $J = \text{Re}\sqrt{K}$, defined as follows: as K is symmetric, there is a unitary (and real) U such that $K = U\text{Diag}(\lambda_1, ..., \lambda_N)U^\dagger$. We define $\sqrt{\lambda_i} = i\sqrt{|\lambda_i|}$ if $\lambda_i < 0$. Then

$$J = U\Big(\text{Diag}(\sqrt{\lambda_1}, ..., \sqrt{\lambda_N})\Big)U^\dagger.$$

This modified algorithm only corresponds to a particular parametrization of the weight matrix and can thus be implemented with the PRIS. The writing of this problem as a function of the coupling matrix eigenvalues (see above) proves that, as long as $\alpha \geq 0$ in the modified algorithm, the ground state of the Ising problem will likely remain unchanged (see Supplementary Figure **4**).

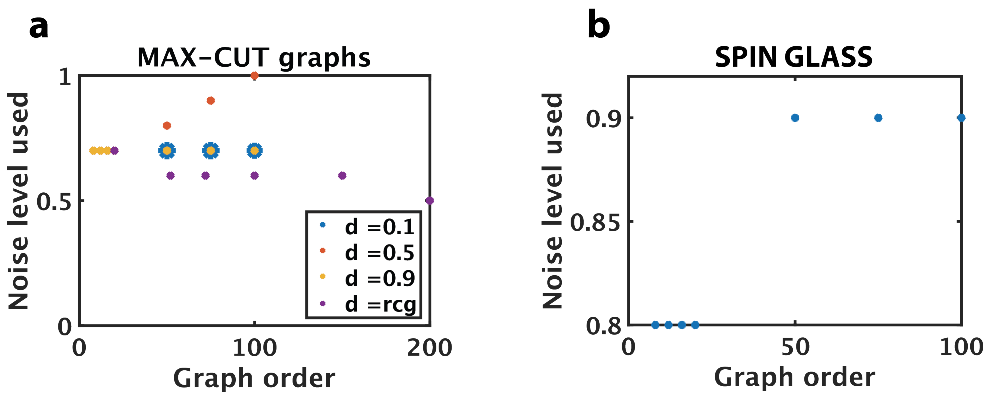

Supplementary Figure 2. **Optimized noise level used in Figure 2 (main text). a:** for MAX-CUT graphs (Figure 2c in the main text). **b:** for spin glasses (Figure 2b in the main text).

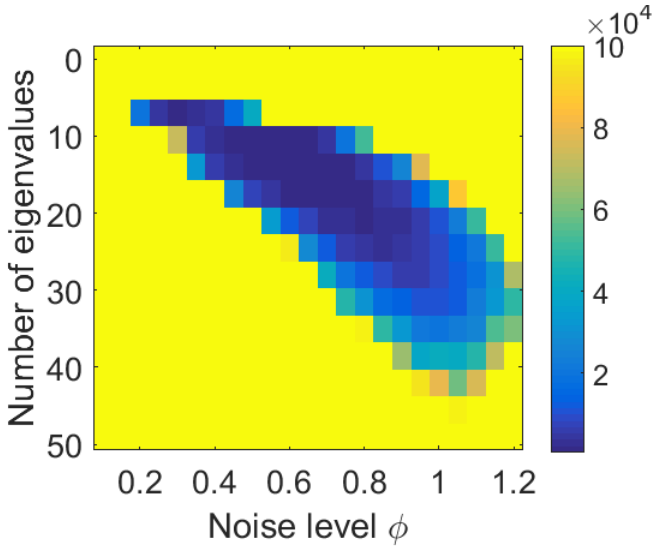

Supplementary Figure 3. **Conjugated influence of eigenvalue dropout and noise levels for finding the ground state.** For each tuple (α, ϕ), the PRIS is run 100 times and $N_{\text{iter},90\%}$ is plotted (the ground state is pre-computed with BiqMac[3]). The colorbar is saturated at 10^5 iterations in order to show the valley of optimal (α, ϕ) values. In this Supplementary Figure, we study a MAX-CUT graph with density $d = 0.5$ and $\Delta_{ii} = \sum_{j \neq i} |K_{ij}|$.

SUPPLEMENTARY NOTE 4: NUMERICAL SIMULATIONS

Evaluating sampling performance and critical parameters on two-dimensional ferromagnetic and infinite-range Ising models

In the main text, we evaluate the performance of the PRIS on sampling the Gibbs distribution of analytically solvable Ising models and estimating their critical exponents. We chose the following two problems:

▷ Two-dimensional ferromagnetic Ising model on a square lattice with periodic boundary conditions. This model was first analytically solved by Onsager[14]. Its energy is defined as

$$H = -\frac{1}{2} \sum_{\langle ij \rangle} \sigma_i \sigma_j, \tag{57}$$

where $\langle ij \rangle$ refers to nearest neighbors (i, j) under periodic boundary conditions (see Supplementary Figure **5**).

▷ Infinite-range Ising model, where each node is connected with a positive coupling of $1/N$ to all its neighbors, including

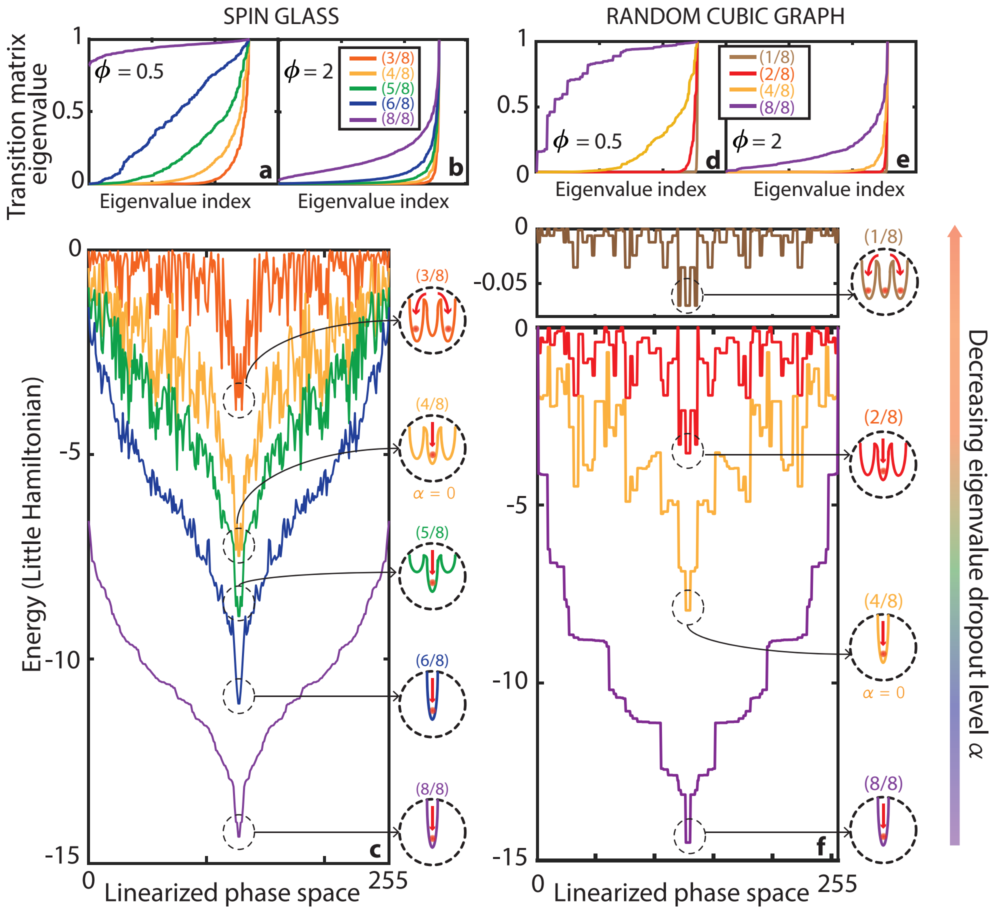

Supplementary Figure 4. **Influence of dropout** (a-c) for a random spin glass and (d-f) for a sample random cubic graph with $N = 8$ and $\phi = 0.5$. (a, b, d, e): Eigenvalue distribution for various noise levels. (c, f): Energy landscape (Hamiltonian) plotted over linearized phase space for various eigenvalue dropout levels α. For this Supplementary Figure, we set $\Delta_{ii} = \sum_j |K_{ij}|$.

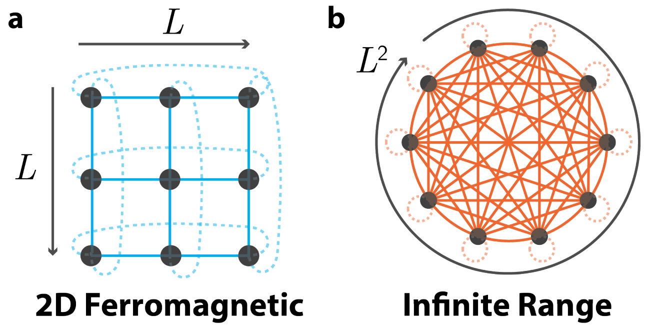

Supplementary Figure 5. **Examples of analytically solvable Ising models studied in our benchmark.** (a) Two-dimensional ferromagnetic Ising model and (b) infinite-range (mean-field) Ising model.

itself. This model can be analytically solved by mean field theory[15]. Its energy is defined as

$$H = -\frac{1}{2N}\sum_{ij}\sigma_i\sigma_j = -\frac{1}{2N}(\sum_i \sigma_i)^2. \tag{58}$$

In order to measure the critical exponents, we need to make sure the free energy at all temperatures is equivalent to that of the corresponding Ising model *at all temperatures*. It has been shown that the free energy of a Little network with coupling matrix

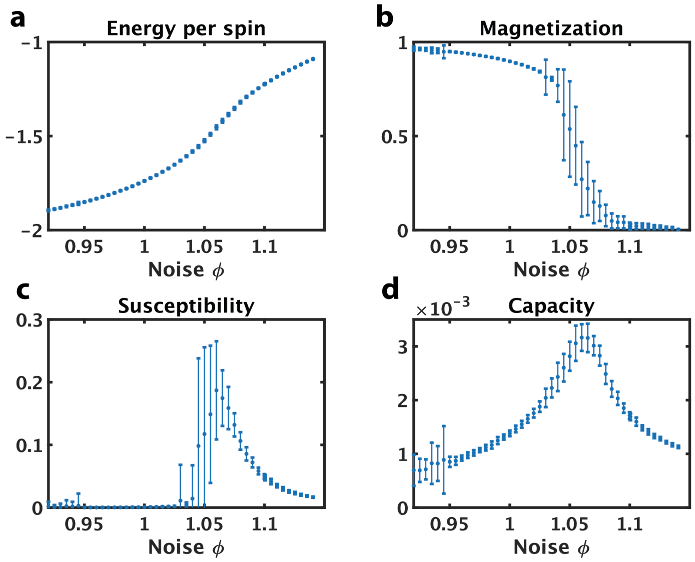

Supplementary Figure 6. **Physical observables of the two-dimensional Ising model obtained with $L = 36$ square-rooted PRIS.**

K equals that of an Ising model defined by the same coupling matrix K, when the coupling weights are configured to learn a set of stable configurations (associative memory)[9]. However, this network has been discarded because of the possible existence of loops between some of its degenerate states, which can result in odd dynamics of the system, if no additional precautions are taken (see, for instance, Supplementary Figure **7**, where this simple algorithm actually converges to the *maximal* energy). We observe that adding a diagonal offset to the coupling, as we do in the square-root version of this algorithm, suppresses these odd dynamics (Supplementary Figure **7**(b)).

The algorithm described earlier to find the ground state of Ising problems could also be used to measure critical exponents, as can be seen in Supplementary Figure **6**. However, there are some complications that arise:

▷ Taking the square root of the coupling matrix prevents the use of symmetry and sparsity to reduce the algorithm complexity. Thus, for large graphs, the time needed to make a single matrix multiplication becomes quite large on a CPU.

▷ Taking the square root also modifies the coupling amplitude. It is now unclear how the temperature of the system should be defined (which is a problem if we want to estimate the critical temperature with the PRIS). From the large-noise expansion of the Hamiltonian, we should define the effective temperature as $T = k^2\phi^2$. However, we observe this definition does not match the theoretical value of the critical temperature of the 2D Ferromagnetic Ising model.

For both Ising problems studied, we perform the following analysis:

▷ We first estimate the Binder cumulant U_4 (see definition in the main text) as a function of the system temperature, for various graph sizes $N = L^2$. The cumulants U_4 plotted for different L intersect at the critical temperature[12] (see Figure 4(a) in the main text).

▷ We then estimate the dependence on the linear dimension L of various observables at the critical temperature and deduce the corresponding critical exponent. This is enabled by the scaling law of observables at the critical temperature[12]:

$$m \sim L^{-\beta_C/\nu_C}, \tag{59}$$
$$\chi \sim L^{\gamma_C/\nu_C}, \tag{60}$$
$$\tau^E_{\text{auto}} \sim L^{z^E_C}, \tag{61}$$
$$\tau^m_{\text{auto}} \sim L^{z^m_C}, \tag{62}$$

where $m, \chi, \tau^E_{\text{auto}}$, and τ^m_{auto} are respectively the magnetization, the magnetic susceptibility, the energy autocorrelation time and the magnetization autocorrelation time[12]. To estimate the critical exponents, we run the algorithm 120 times for 10^5 iterations, with random initial conditions, at the critical temperature for each L and fit these observables with a power law in L.

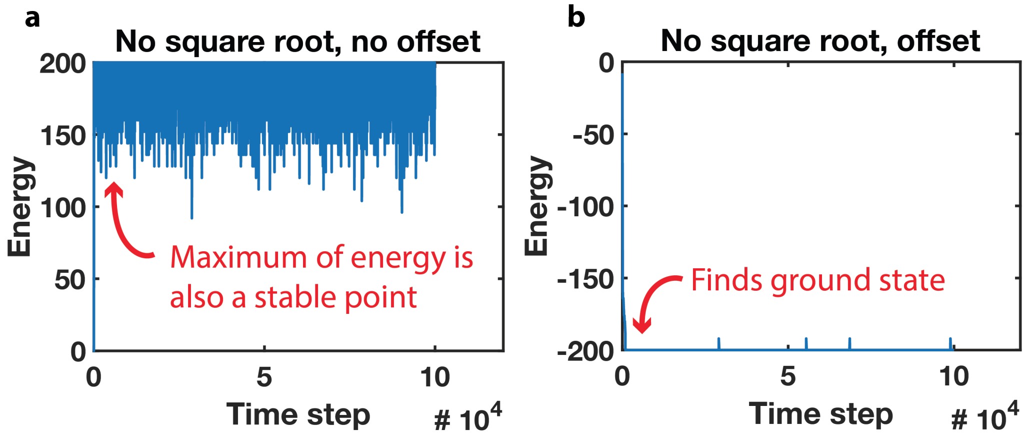

Supplementary Figure 7. **Comparison of different versions of the algorithms on the 2D Ferromagnetic Ising Model.** (a) With no offset, the no square root algorithm (defined by the following regime of operation of the PRIS: $C = K$) results in convergence to the state with maximal energy, for some runs (with random initial conditions). (b) Adding an offset to the same regime of operation cancels this behavior and the algorithm always converges to the ground state after some time.

Algorithm	β_C	R^2	γ_C	R^2	z_C^E	R^2	z_C^m	R^2
**2D ferromagnetic Ising model**								
MH	0.1257	0.992	1.735	0.998	1.393	0.993	2.068	0.998
PRIS	0.1194	0.972	1.867	0.990	1.860	0.977	2.023	0.994
**Infinite-range Ising model**								
MH	0.5245	0.997	1.011	0.999	0.920	0.999	0.924	0.999
PRIS	0.5650	0.999	0.922	0.999	0.914	0.999	0.886	0.999

Supplementary Table 2. **Summary of critical exponents measured with the PRIS and MH.** R^2 is the coefficient of determination of each power law fitting.

The estimates of all observables are obtained by taking every τ_{auto}^E generated samples, where τ_{auto}^E is the energy autocorrelation time, and dropping the first 10% of the data (arbitrary burn-in or equilibrium time). Our findings are summarized in Supplementary Table **2**, Supplementary Figures **8** and **9**, and are benchmarked versus the Metropolis-Hastings algorithm, which is summarized in Refs.[12,16,17].

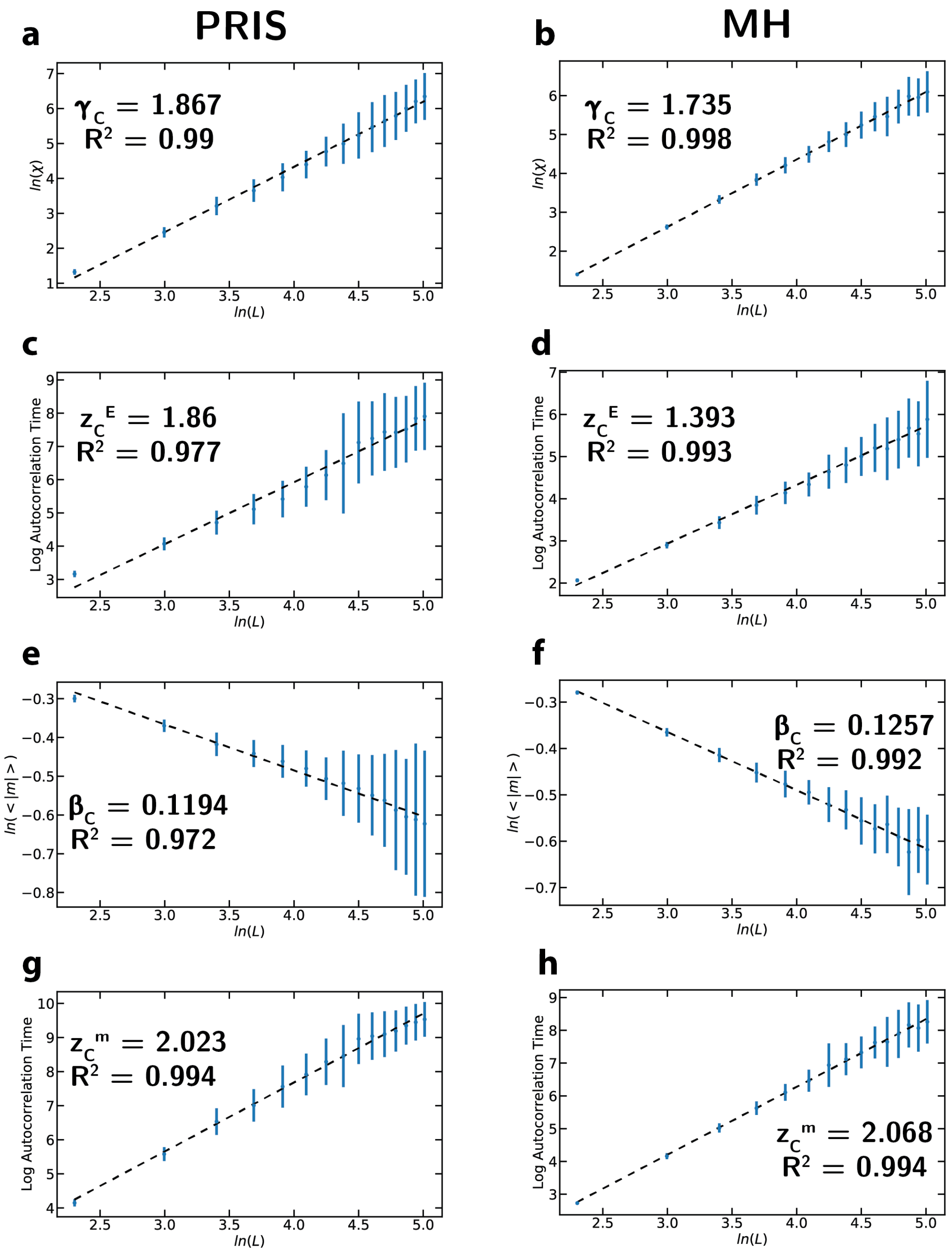

Supplementary Figure 8. **Probing the critical exponents of the 2D Ferromagnetic Ising model.** Fits are shown with the resulting critical exponent for the PRIS **(a, c, e, g)** and the MH **(b, d, f, h)** algorithms for the susceptibility **(a-b)**, energy autocorrelation time **(c-d)**, magnetization **(e-f)**, and magnetization autocorrelation time **(g-h)**.

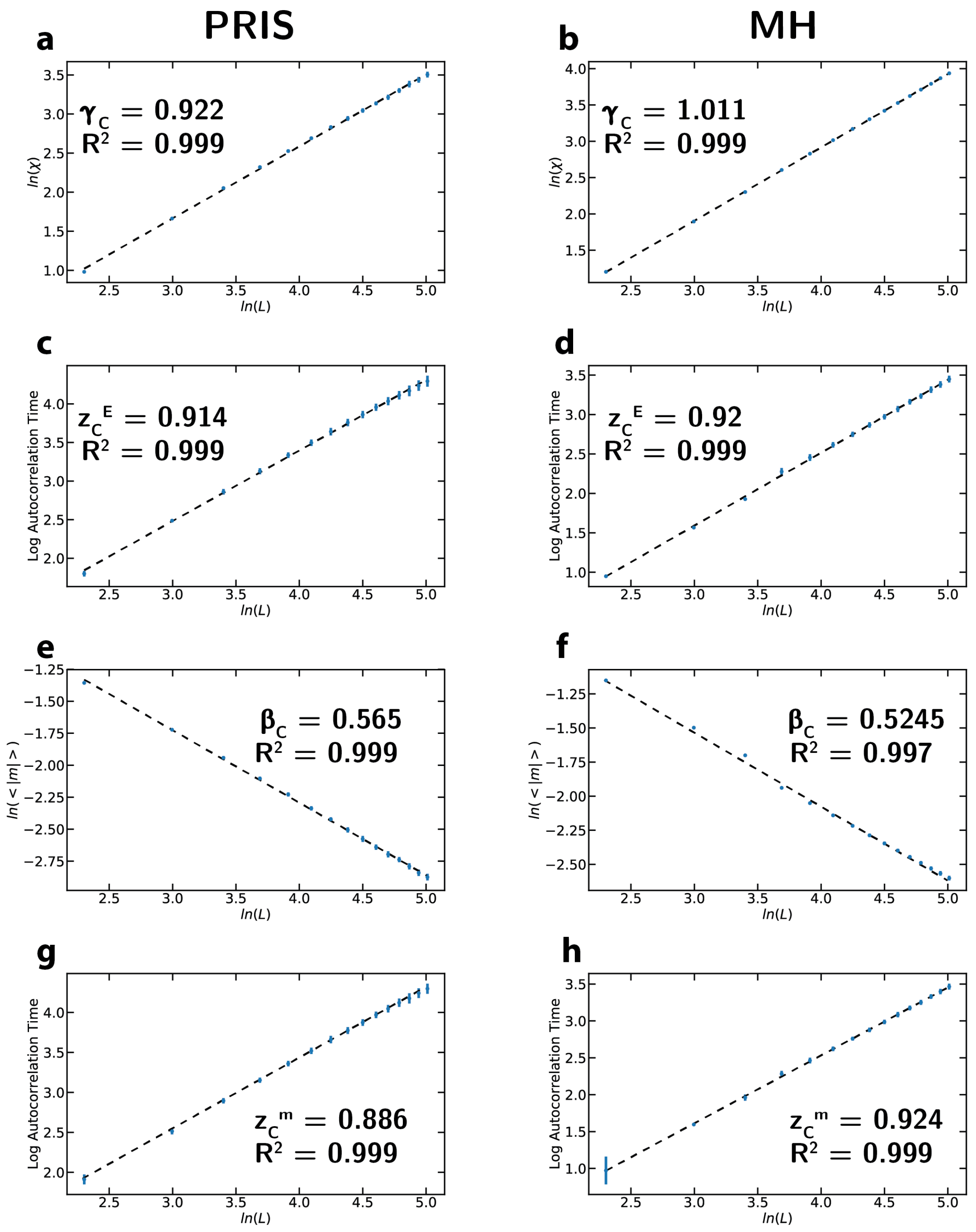

Supplementary Figure 9. **Probing the critical exponents of the infinite-range Ising model.** Fits are shown with the resulting critical exponent for the PRIS **(a, c, e, g)** and the MH **(b, d, f, h)** algorithms for the susceptibility **(a-b)**, energy autocorrelation time **(c-d)**, magnetization **(e-f)**, and magnetization autocorrelation time **(g-h)**.

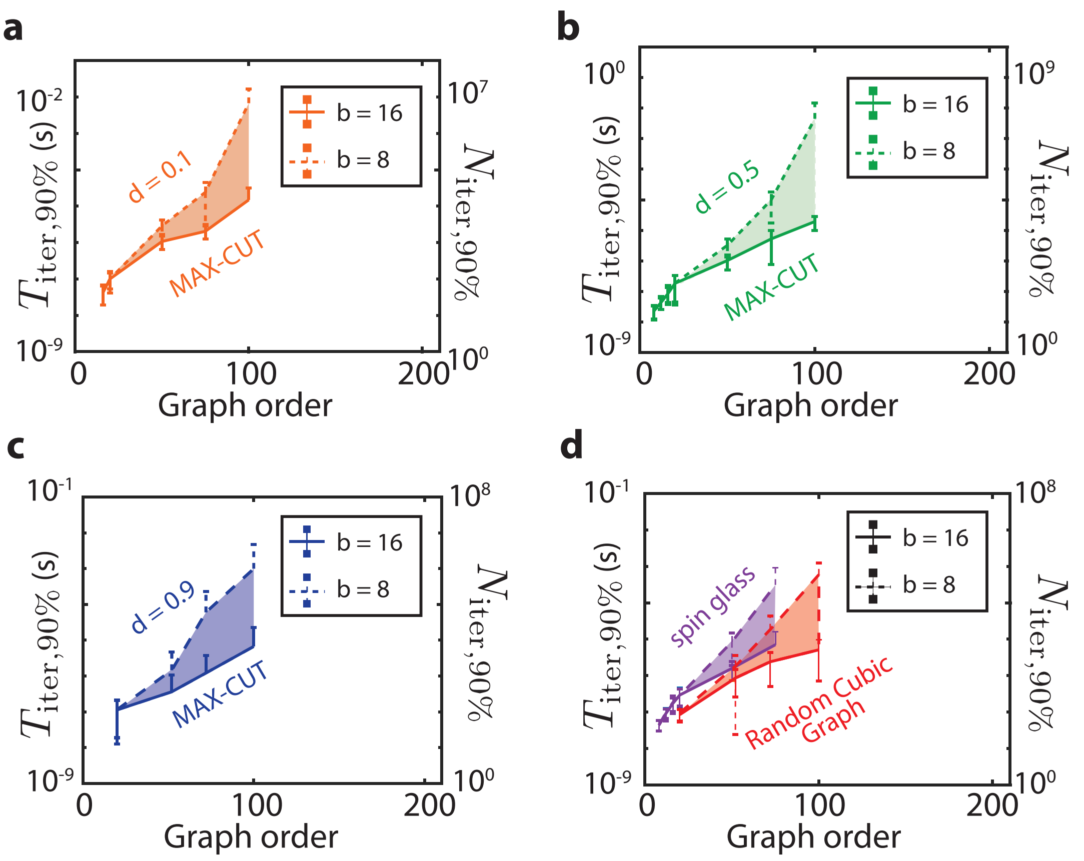

Supplementary Figure 10. **Simulated scaling of PRIS on a cascaded array of programmable MZIs.** Number of algorithm steps to reach a ground state with a probability of 90% is plotted as a function of the graph order for various graph topologies: MAX-CUT graphs with densities $d = 0.1$ **(a)**, $d = 0.5$ **(b)**, and $d = 0.9$ **(c)**, Random Cubic Graphs (RCG), and spin glasses **(d)**.

SUPPLEMENTARY NOTE 5: SCALING OF THE PRIS PERFORMANCE ON SEVERAL PHOTONIC ARCHITECTURES

Cascaded arrays of programmable Mach-Zehnder interferometers (MZI)

In Supplementary Figure **10**, we examine the influence of the bit accuracy of the setting of the phases on the performance of the machine. This can describe any system where the matrix is encoded by a cascaded array of programmable MZIs such as[13,18]. We assume the phase can be set with b-bit accuracy, which means that when setting phase θ_m (resp. ϕ_m) on the PNP, we actually draw from a uniform distribution $\theta \in [\theta_m - \frac{2\pi}{(2\pi)^b}; \theta_m + \frac{2\pi}{(2\pi)^b}]$ (resp. $\phi \in [\phi_m - \frac{2\pi}{(2\pi)^b}; \phi_m + \frac{2\pi}{(2\pi)^b}]$).

Another way to describe the bit accuracy of the PNP is through the bit-accuracy of the voltage setting[13]. The phase-voltage relation can be approximated by a quadratic dependence: $\theta = 2\pi \left(\frac{V}{V_{2\pi}}\right)^2$ where $V_{2\pi}$ is the voltage setting to achieve 2π modulation[19]. If we assume the voltage is set with b_V accuracy, then the actual voltage is drawn from a (uniform) distribution over $[V - \frac{V_{2\pi}}{2^{b_V}}; V + \frac{V_{2\pi}}{2^{b_V}}]$. This translates to the phase accuracy as:

$$\theta \pm \Delta\theta = 2\pi \left(V \pm \frac{V_{2\pi}}{2^{b_V}}\right)^2 / V_{2\pi}^2 \tag{63}$$

$$= \theta \pm \frac{\sqrt{2\pi\theta}}{2^{b_V - 1}} + \frac{2\pi}{4^{b_V}} \tag{64}$$

We can safely neglect the last term and we notice that the worst case scenario corresponds to $\theta \sim 2\pi$, for which $b_V - 1 = b_\theta$. A rule of thumb results: a b_V-bit accuracy of the PNP on its voltage setting corresponds to a $(b_V - 1)$-bit accuracy of the PNP on its phase setting. Inversely, a b_θ-bit accuracy of the PNP on its phase setting corresponds to a $(b_\theta + 1)$-bit accuracy of the PNP on its voltage setting.

Static sources of noise could be a significant bottleneck in scaling the PRIS to large $N \sim 100$ graph orders. For instance,

a static noise on the phase setting of an array of MZI will result in a static error on the effective coupling between spins, thus reshaping the Hamiltonian landscapes, which could impact the algorithm efficiency. We simulate the algorithm performance as a function of the graph order N for phase resolutions of $b_\theta = 8$ and 16 bits. The resulting time on a GHz photonic architecture to find the ground state with 90% chance, $T_{\text{iter},90\%}$, is also shown in Supplementary Figure **10**. While a 16-bit phase resolution does not impact the algorithm performance (with scaling results comparable to an ideal photonic network, see main text), an 8-bit phase resolution may increase the required number of algorithm steps by one to two orders of magnitude, depending on the graph order and topology (while still outperforming other photonic systems on a GHz architecture, such as[20,21]). Thus, the reduction of static noise is of paramount importance in the realization of the PRIS on large-scale static photonic networks.

Optical Neural Networks based on Photoelectric Multiplication

For larger graphs $N \sim 10^3 - 10^6$, one could resort to recently-proposed large-scale optical neural networks based on photoelectric multiplication[22]. By encoding both matrix weights C_{ij} and input signals $S_i^{(t)}$ into optical (time) domain, the measured output is added a Gaussian noise term with amplitude defined by the number of photons per Multiply And Accumulate (MAC):

$$S_i^{(t+1)} = \text{Th}_\theta \left(\sum_j C_{ij} S_j^{(t)} + w_i^{(t)} \frac{||C||||S^{(t)}||}{N^{3/2}} \frac{\sqrt{N}}{\sqrt{n_{\text{mac}}}} \right). \tag{65}$$

For the various problems we study in this paper, the working standard deviation is usually ~ 1. This corresponds to a working n_{mac} of

$$n_{\text{mac}} \sim N \frac{||C||^2||S^{(t)}||^2}{N^3} \tag{66}$$

We can evaluate the corresponding total energy consumption per matrix multiplication for 10 random spin glasses. We get $n_{\text{mac}} \sim 4$ (resp. ~ 15) for $N = 100$ (resp. $N = 1,000$). Smaller working n_{mac} will be required for sparser graphs, since $||C||$ is smaller. The corresponding SNR scales as $\sim n_{\text{mac}}$ and the total energy is $N^2 n_{\text{mac}} \sim 6.2 \pm 0.35$ fJ/matrix multiplication (resp. 1.9 ± 0.5 pJ/matrix multiplication).

There are many attractive features of these networks for the implementation of large-scale PRIS:

- ▷ This architecture naturally leverages quantum noise which perturbs the output as is required for the good execution of the PRIS. The noise level can be tuned by changing the number of photons per MAC which is proportional to the SNR.
- ▷ The non-linear function is executed in electronic domain, which allows a lot of flexibility on its implementation and reconfiguration (the threshold function required for the good operation of the PRIS would be straightforward to implement), while optical nonlinearities working at low-power have not been demonstrated so far.
- ▷ This architecture is in principle scalable to very large number of spins $N \sim 10^6$.

Free space optical Neural Networks

Since the PRIS relies on a static transformation, the use of free-space optical neural networks[23,24] with 3D printed masks or reconfigurable SLMs is another option to achieve PRIS with $N \sim 10^6$ neurons. The analysis we made on the influence of heterogeneities (see Supplementary Figure **10**) remains relevant here. A set of lens - mask - lens would realize the matrix multiplication on the signal $S^{(t)}$ encoded in optical domains, while the coupling matrix C is encoded in the mask transmission. The speed of such a free-space architecture would only be limited by the photodetector (typically ~ 10 THz) and modulation (typically ~ 1 kHz for SMLs, $\gtrsim 1$ GHz for on-chip modulators) bandwidths.

Comparison table of various heuristic algorithms and architectures

Since the PRIS essentially relies on fast vector-to-fixed matrix multiplication, it can be implemented efficiently on various photonic and parallel electronic hardware architectures, such as FPGAs. We summarize in Supplementary Table **3** the projected performance of various heuristic algorithms (MH and PRIS) running on several hardwares (both electronics and photonics). We observe that, while photonics potentially allows the fastest clock for such algorithms and a competitive scaling factor, FPGAs can achieve similar clocks and require much less engineering (they can be bought off the shelf and are easily reconfigurable). To

Algorithm	Algorithm step	Architecture	Algorithm step time estimate with $N = 100$	Time Complexity
MH	Sequential	CPU	~ 1 ms*	$O(N^2)$
PRIS	Parallelizable	CPU	$\sim 10 - 100\mu$s*	$O(N^2)$
		FPGA	$\sim 5 - 100$ns**	$O(N^2/M)$
		MZI network[13,18]	$\sim 0.1 - 1$ns	$O(N)$
		Free space optics[21,25]	modulation bandwidth limited (SLM $\sim$ 1ms, electro-optic modulators $\sim 0.1 - 1$ns)	$O(1)$

Supplementary Table 3. **Comparative table of projected performance of various heuristic algorithms implemented on various architectures.** Time complexity is given as a function of the total number of spins N. Regarding clock estimates: * Estimate run on a 2.7 GHz Intel Core i5 with Matlab, ** Estimate run on a Xilinx Zynq UltraScale+ MPSoC ZCU104 with a systolic array architecture. See Supplementary Note 7 for more details.

demonstrate our point, we perform a proof-of-concept experiment on a FPGA board, whose results are shown in Supplementary Note 7.

If photonic architectures can significantly reduce the time complexity of the algorithm step by performing massive multiplexing[25], it must be noted that we are neglecting time overhead such as fabrication, etc. Also, some free-space architectures, such as SLM, are typically slow to reconfigure (on the order of 1ms), thus only being relevant for multiplying very large matrices.

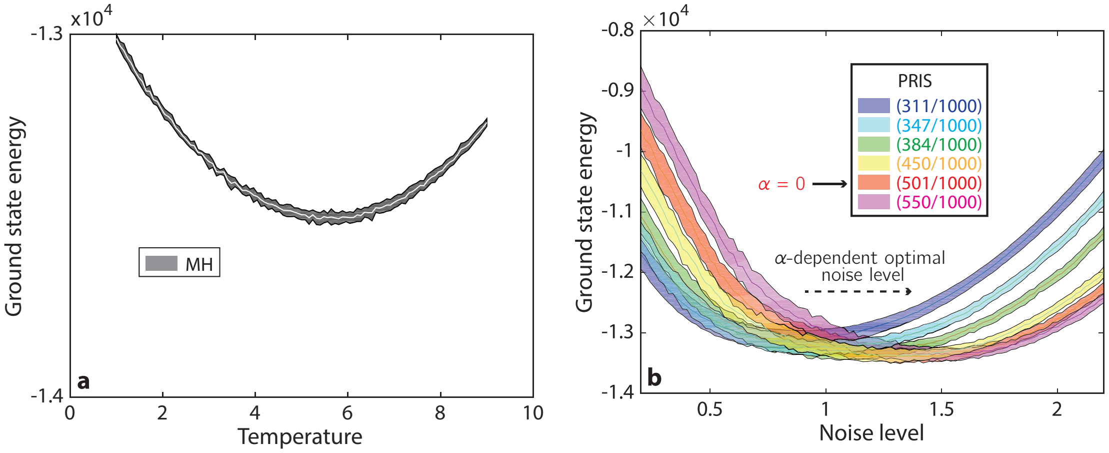

Supplementary Figure 11. **Benchmarking the PRIS versus MH on large spin glasses $N = 1000$.** For both PRIS and MH, the algorithm is ran 100 times for 10,000 iterations at each temperature / noise level. The line shows the average ground state energy over 100 runs, and the shaded area corresponds to $\pm$ the standard deviation. PRIS is ran for various dropout levels corresponding to (N_{eig}/N) eigenvalues (see legend). Results for MH and PRIS at the smallest eigenvalue dropout level (311/1000) are also averaged over the 10 random spin glasses. Results for the PRIS at all others eigenvalue dropout levels are only averaged over spin glass 1 (see discussion). For this study, we choose $\Delta_{ii} = |\sum_j K_{ij}|$.

SUPPLEMENTARY NOTE 6: COMPARISON OF THE PRIS TO SEVERAL (META)HEURISTICS

Benchmarking versus Metropolis-Hastings on large spin glasses $N \sim 1000$

In order to evaluate the performance of the PRIS on larger graphs, for which exact solvers typically fail, we benchmark the PRIS against MH for a set of 10 random spin glasses (whose couplings are randomly chosen from a uniform distribution in the interval $[-1, 1]$). First, we notice that the behavior probed by the PRIS and MH is very similar over the 10 spin glasses. The temperature/noise-dependent mean ground state energy found by MH and the PRIS is shown in Supplementary Figure **11**. In particular, we see that the PRIS achieves mean ground state energies similar to MH. As a reminder, a heuristic mapping between the temperature from statistical physics and the effective temperature (noise level) of the PRIS is $T \sim (k\phi)^2$. These graphs orders are large enough, so that standard exact solvers are taking a long time to find the ground state. For instance, we ran BiqMac on spin glass 1 on their online submission server[3] for three hours. The algorithm could only find an approximate ground state which was outperformed by MH, PRIS-A, and SA.

We also observe that the PRIS shows an α-dependent optimal noise level, which increases with α. For spin glass 1, the absolute lowest ground state energy is obtained for $\alpha = -0.2$, i.e. (450/1000) eigenvalues. This hints at the necessity of optimizing the hyperparameter α around $\alpha \sim 0$ when running the PRIS on large graphs.

PRIS-A: a proposed metaheuristic variation of the PRIS

A route to achieving systematically low energy states is to design metaheuristics, i.e. master strategies guiding the search for an optimal ground state. Simulated Annealing (SA)[26] is one of such algorithms, derived from Metropolis-Hastings. Let us reminder the reader of one possible implementation of this algorithm with the widely used geometric schedule for the temperature[26–29]:

Start from random initial state
Choose initial temperature T_i and geometric factor $\lambda < 1$
$T \leftarrow T_i$
for all $i \in \{1, ..., N_{\text{alg, iter}}\}$
  $T \leftarrow \lambda T$
  for all $j \in \{1, ..., N_{\text{iter per temp.}}\}$
    Update state according to MH acceptance rule at temperature T
  end for
end for

Algorithm 1: Simulated annealing algorithm with a geometric schedule.

Instance	PRIS	MH	PRIS-A	SA	BiqMac
spin glass 1	-13,716	-13,796	-13,795	-13,814	-13,746
spin glass 2	-13,632	-13,736	-13,716	-13,754	N.C.
spin glass 3	-13,717	-13,777	-13,760	-13,796	N.C.
spin glass 4	-13,678	-13,768	-13,781	-13,798	N.C.
spin glass 5	-13,732	-13,755	-13,767	-13,782	N.C.
spin glass 6	-13, 752	-13,828	-13,804	-13,832	N.C.
spin glass 7	-13,723	-13,792	-13,758	-13,800	N.C.
spin glass 8	-13,731	-13,769	-13,781	-13,783	N.C.
spin glass 9	-13,711	-13,810	-13,798	-13,817	N.C.
spin glass 10	-13,754	-13,846	-13,822	-13,855	N.C.

Supplementary Table 4. **Summary of benchmarking PRIS and PRIS-A against MH and SA.** For both PRIS and MH, the algorithm is ran 100 times for 10,000 iterations at each temperature / noise level. The table shows the absolute lowest ground state energy recorded. For PRIS-A and SA, the algorithm is ran 100 times with $N_{\text{alg, iter}}$ temperature increments (as given by Supplementary Equation (67)) and $N_{\text{iter per temp.}} = 100$. For PRIS-A (resp. SA), the initial noise level (resp. temperature) is $\phi_i = 50$ (resp. $T_i = 5,000$), the final noise level is $\phi_f = 0.1$ (resp. $T_f = 0.01$) and the temperature geometric factor is $\lambda = \sqrt{0.991}$ (resp. $\lambda = 0.991$). For PRIS-A, the eigenvalue dropout level is taken to be $\alpha = 0$, corresponding to (501/1000) eigenvalues. For BiqMac, the algorithm ran for three hours (time limit on the BiqMac online job submission platform). N.C. = Non Computed.

This naturally inspires a metaheuristic based on the PRIS, which we call Photonic Recurrent Ising Simulated Annealing (PRIS-A):

Start from random initial state
Choose initial noise level ϕ_i and geometric factor $\lambda < 1$
$\phi \leftarrow \phi_i$
for all $i \in \{1, ..., N_{\text{alg, iter}}\}$
  $\phi \leftarrow \lambda\phi$
  for all $j \in \{1, ..., N_{\text{iter per temp.}}\}$
    Update state according to PRIS acceptance rule at noise level ϕ
  end for
end for

Algorithm 2: Photonic Recurrent Ising Simulated Annealing (PRIS-A) algorithm.

Let us note a couple of peculiarities:

- ▷ The noise level from the PRIS is related to an effective temperature via $T \sim \phi^2$. Thus, one should compare SA ran with a geometric factor λ to PRIS-A with a geometric factor $\sqrt{\lambda}$.
- ▷ In the PRIS-A algorithm, the eigenvalue dropout level α is also a degree of freedom. One could thus, in principle, also simulate the annealing of the eigenvalue dropout level, thus affecting the ground state search dimensionality. A comprehensive study of this new class of algorithms goes beyond the scope of this work.
- ▷ For given initial and final noise levels/temperatures and geometric factors, one can determine the number of temperature increments $N_{\text{alg, iter}}$ with the formula:

$$N_{\text{alg, iter}} = \frac{\log \phi_f - \log \phi_i}{\log \lambda}. \tag{67}$$

- ▷ λ is typically chosen to be smaller but close to 1, in order to mimic adiabatic temperature variations. We verify that both for SA and PRIS-A, $\lambda > 0.98$ yields consistently low energy ground states, with no particular amelioration when increasing λ (and scaling the number of temperature increments $N_{\text{alg, iter}}$).

The initial and final noise levels chosen for PRIS-A are $\phi_i = 50$ and $\phi_f = 0.1$. The initial and final temperatures chosen for SA are $T_i = (2 * 0.5877 * \phi_i)^2 \sim 3454$ and $T_f = (2 * 0.5877 * \phi_f)^2 \sim 0.01$. We observed no significant variation on the minimum ground state energy found for $\lambda > 0.99$ and ran each algorithms with a rate of $\lambda = 0.991$. The performance of the various algorithms we implement is shown in Supplementary Table **4**. MH yields lower ground state energies than PRIS on average of 0.53%. However, PRIS-A can outperform PRIS by a similar amount, lowering energies on average of 0.46%. This is a larger performance enhancement than SA to MH (0.11% decrease of ground state energy). Then, on average, SA outperforms PRIS-A by 0.18%. We expect optimization of the eigenvalue dropout level α (and simulated annealing on this parameter) to further enhance the performance of PRIS and PRIS-A.

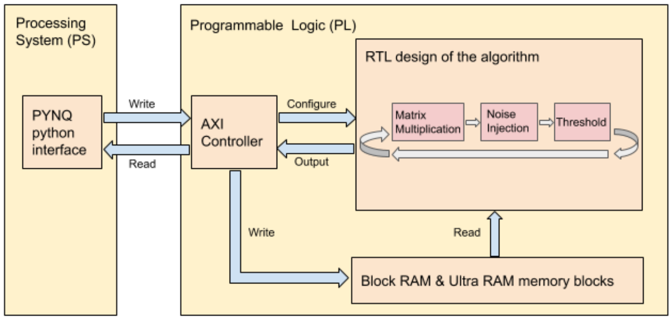

Supplementary Figure 12. High level architecture of the design.

SUPPLEMENTARY NOTE 7: IMPLEMENTATION OF THE PRIS ON FPGA

Architecture

The PRIS algorithm is primarily designed for future photonic implementations. However, both photonic chips and FPGAs (Field Programmable Gate Arrays) share parallel processing capability. Thus, it is worthwhile to first demonstrate the performance of the algorithm on an FPGA board.

We have implemented the algorithm on Xilinx Zynq UltraScale+ multiprocessor system-on-chip (MPSoC) ZCU104 evaluation board based on the Pynq framework. The high-level synthesis, place, and route have been performed by Xilinx Vivado 2018.3 design suite. Zynq and Zynq Ultrascale+ devices integrate a multi-core processor (ARM Cortex-A9) and programmable logic (FPGA) in the same circuitry. PYNQ is an open-source project released by Xilinx that enables Python Productivity for Zynq devices. It incorporates the open-source Jupyter notebook infrastructure to run an Interactive Python (IPython) kernel and a web server directly on the ARM processor of the Zynq device. It also provides extensive hardware libraries (overlays) and APIs which enable easier and faster programming of FPGA.

For our application, the preprocessing and preparation of data is performed in a Jupyter notebook using Python. Once ready, we transmit the data and write to memories on FPGA through AXI interface. After running the recurrent algorithm for a number of cycles on FPGA, the final state vector is transmitted back for data analysis like energy calculation and correctness verification. In this manner, the same hardware configuration could be easily reconfigured and run different problems efficiently. Noise generation and post-processing (energy calculation, and more generally extracting observable) could also be run directly on the FPGA in future versions of this implementation.

The overall high level architecture is shown in Supplementary Figure **12**. We make use of the address-mapped AXI interface to encode different operations (e.g. Block RAM (BRAM) addressing, loop setting, result selection) into different addresses. AXI controller loads matrix, noise and threshold data into BRAMs, then sets up the initial state vector and starts the computation. The loop module is a finite-state-machine which reads the data from BRAM, adds noise, compares with threshold and then updates the state vector at every loop step. The final state vector and clock information is transmitted back through AXI.

We emphasize the following considerations regarding our implementation:

- ▷ The bottleneck of the performance implementation is to read input matrix data from memory. The ZCU104 board has 312 BRAM blocks, with each block transmitting a maximum of 72 bits per clock cycle when configured under true-dual-port mode. It also has 94 UltraRAM (URAM) blocks with each block transmitting a maximum of 144 bits per clock cycle. Thus the total maximum data rate it could read from BRAM and URAM is 36288 bits per clock cycle. For our current implementation ($N < 100$) we could assume we have enough memory, but as the problem size increases this becomes a major limitation.

- ▷ We multiply an N-by-N b bit matrix (b being the bits we choose to represent the Ising problem data) with an N-by-1 1-bit state vector, so each data in the result is a conditional sum of one row of matrix C based on the value of the state vector. We choose to implement a binary tree for speeding up the sum as shown in Supplementary Figure **13**. At each time step, a certain number of rows of the matrix is loaded onto the leaves of the tree, and then gets propagated to next level based on the value of the state vector, and adds all up thereafter. The result of the sum should propagate to the root after a delay of clock cycles equal to the height of the tree.

The loop is carefully pipelined, in which reading the matrix takes $\frac{N}{2b}$ clock cycles, multiplication (binary tree addition) takes $\log_2 N$ cycles, while noise injection and thresholding are done in a single clock cycle. Details of the complexity is discussed

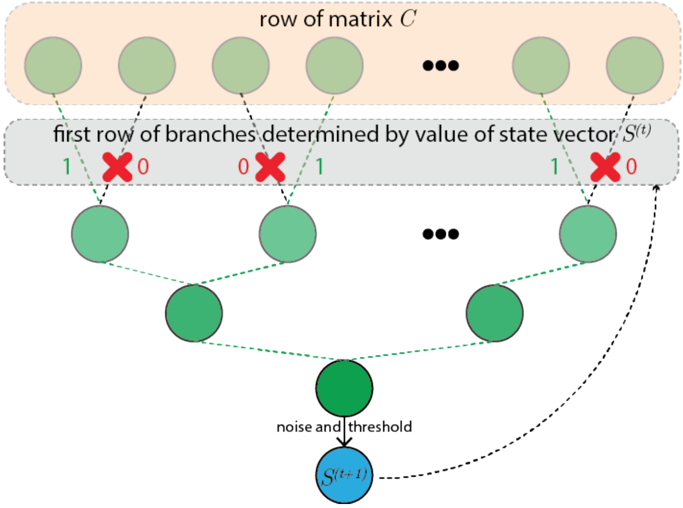

Supplementary Figure 13. **Binary tree architecture for matrix-to-vector multiplication.** A row of matrix C is read from memory to the top leaves of the binary tree. The branch connecting the top leaves to the next tree layer are either active or not, depending on the value of the current state vector (0 or 1).

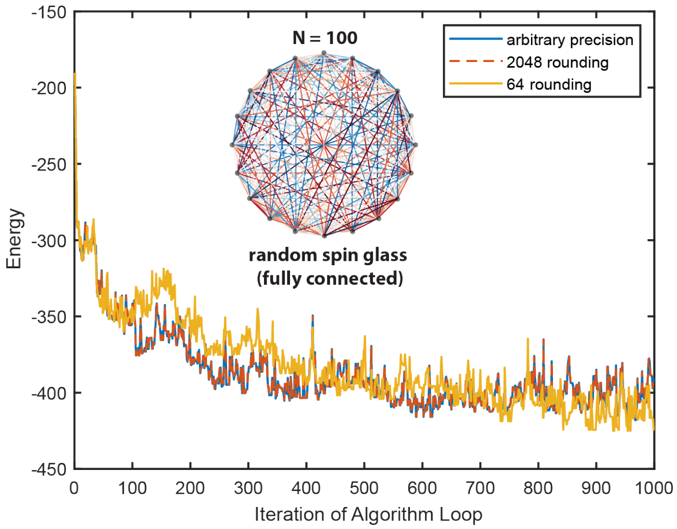

Supplementary Figure 14. **Performance comparison for various rounding values.**

in the next session. We are only running integer arithmetic on the FPGA, so to adapt the original algorithm which runs on float points, we need to scale the parameters (matrix, noise, and threshold) and then round them to integers. The scaling factor and bit length to represent each data needs to be carefully designed.

Note that the only difference between our implemented algorithm on the FPGA and the ideal algorithm is that we round our values to a specific bit depth. Specifically, we store the matrix values, noise, and threshold values only to a specific bit precision. This originates from memory constraints on the FPGA, but it is reasonable to suspect that this rounding affects the performance of the algorithm. To test the effects of this change, we implemented both our original algorithm and the version with rounding on MATLAB, testing various bit depth with varying input, as shown in Supplementary Figure **14**. We found that, for a matrix of size $N = 100$ by 100, rounding after multiplying by 64 or even 32 performed just as well as using arbitrary bit precision (Matlab's default double-precision floating point). Specifically, for a variety of matrix inputs, on average using 64 and 32 roundings reached states of essentially equivalent energy to the arbitrary precision. Over 1000 trials each with a different input matrix, 1000 iterations each (i.e. 1000 matrix multiplications and noise additions and thresholding for each trial), on average our normal algorithm reached a minimum energy of -408.63, whereas the 32 rounding reached -409.08, and the 32 bit reached -409.17.

Complexity

In discussing the complexity of our implementation, we must note the dependence on the particular resources of a given FPGA. Here we will discuss the complexity of a single step of our algorithm, consisting of a single matrix multiplication followed by noise addition and threshold comparison. Since the clock speed of any FPGA is variable, we give the complexity in terms of clock cycles.

We focus on computing the complexity of the matrix multiplication, since it dominates the three steps. To perform this matrix multiplication, we must read the entire matrix from memory and feed it to a system of binary trees. Suppose we store each matrix entry using b bits, and that we are working with an $N \times N$ size matrix. In our FPGA, we can read 64 bits from each BRAM block per clock cycle, so one limiting factor is the total number k of BRAM blocks we can use concurrently. Note that we are also limited by the maximum number R of bits that can be held in registers, or the working memory, of our FPGA. This allows us to compute a complexity bound.

Assuming the FPGA can read M bits from each BRAM block per clock cycle, it will take the FPGA at least $\frac{N^2 b}{M}$ cycles to read the entire matrix. According to the above considerations, $M = 64k + 144u$, where k (resp. u) is the number of used BRAM (resp. URAM). In our current implementation, we only used BRAM memory so $u = 0$. Alternatively, we are also limited by the amount of data we can work with at a time, R. To complete this matrix multiplication, we must store a binary tree corresponding to each row. The result of these binary trees is a vector of N values to be sent to the noise addition step. Thus the total amount of data we will need to work with in this computation is $2N^2 b$ bits, which will take at least $\frac{2N^2 b}{R}$ cycles to pass through the registers of the FPGA.

To finish the analysis, note that we may feed new data into the binary trees before the original data has fully propagated through. Thus the only computation time outside of that required to load the matrix is the time for the final data to propagate through the binary trees. The size of our binary trees can be determined by considering the minimum of the amount of data we can load at a time, $M = 64k$, and the amount of data (in bits) we can work with at a time, R. Note that we never need a binary tree larger than the size of a row of the matrix, and that the binary tree takes up twice the space of the data inputted into it. Then the size of our binary tree is $B := \min(\frac{64k}{b}, \frac{R}{2b}, N)$, and it takes on the order of $\log_2 B$ cycles to propagate through. Then, incorporating the time to load the matrix, the total number of cycles for a single algorithm step is on the order of

$$\text{number of clock cycles per PRIS algorithm iteration} = \max\left(\frac{2N^2 b}{R}, \frac{N^2 b}{64k}\right) + \log_2 B.$$

Lastly, considering noise addition and thresholding, note that this only involves reading $2Nb$ more bits from bram. The actions of noise addition and thresholding themselves only take two clock cycles, and the time and space necessary to deal with these extra $2Nb$ bit is negligible in complexity compared to that necessary to deal with the $N^2 b$ bits from the matrix. So these two steps do not change the overall complexity. We also need to load values into the BRAM in the first place, but we do not include this process in the analysis. Thus the overall complexity of a single iteration of our algorithm is $O\left(\max(\frac{2N^2 b}{R}, \frac{N^2 b}{64k}) + \log_2 B\right)$.

The discussion above is under the assumption that for large size problems ($N > 100$), board registers are not enough for holding all the data in the matrix. For small size problem, we could alternatively use registers and do the multiplication in one clock cycle, then the major time consumption would be the binary tree addition, which took $\log_2 B$ clock cycles.

For problems with larger size, we store matrix in BRAM as mentioned above. We could plot the complexity (Supplementary Figure **15**) discussed above under different assumptions: (1) Using all of the memory blocks on the FPGA (Supplementary Figure **15**(a) and (b)), which is optimal but takes time to implement for every problem size. (2) For most reasonably-sized problems, we can assume a linear scaling of memory blocks, i.e. $M = rNb$, which is sub-optimal for a given N but easier to reconfigure for various N allowing this approximation (we can switch between different problem sizes N by changing the width of BRAM IPs in our design). We also assumed that R is large, thus not being the limiting factor of the computation.

Experiment

In this experiment, we tested the correctness and runtime for a problem size of $N = 100$ under a 300 MHz clock. We used 16 bits to encode each data in matrix, noise and threshold. A total of $k = 5$ BRAM IPs are instantiated in the design, each with data length of 1600 bits, corresponding to one row of the matrix. (In this design we are using 71% of BRAM blocks on our board.)

The experiment runs as follows:

- ▷ Original Test: First we generate a test case running on float numbers in Jupyter notebook using Python. We run the test for 200 clock cycles and record the state vector and energy of each iteration.

- ▷ Scale the problem: We scale up the matrix, noise and threshold by a factor of 128 and round to integers. We run the scaled test for 200 clock cycles and plot the energy array together with the original test to check the accuracy of the algorithm

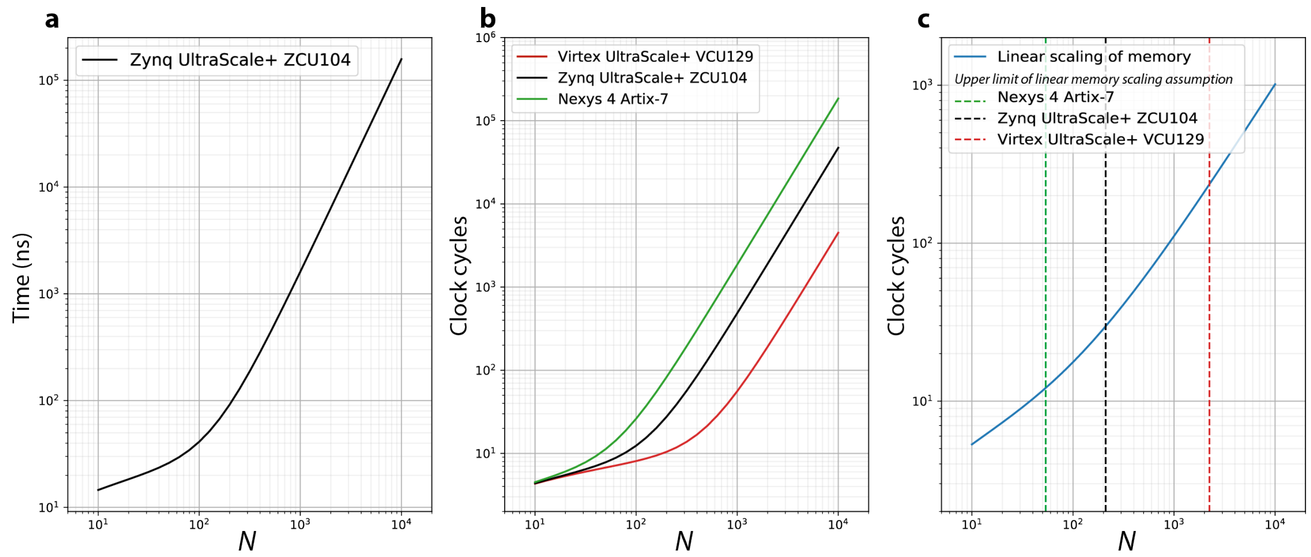

Supplementary Figure 15. **Complexity estimate of a PRIS single algorithm iteration, implemented on FPGA. a:** Graph showing the estimated time consumption on the current ZCU104 board, given that were using all of the memory blocks and under a clock of 300MHz and 16 bit encoding. **b:** Comparison of three different FPGA boards with different memory resources. **c:** Assume linear scaling of memory, i.e. $M = rNb$, here we let $r = 10$ (read 10 rows of matrix at a time). The vertical lines indicate the limit of each FPGA. For our ZCU104 board, the maximum problem size under the linear scaling assumption is $N = 212$.

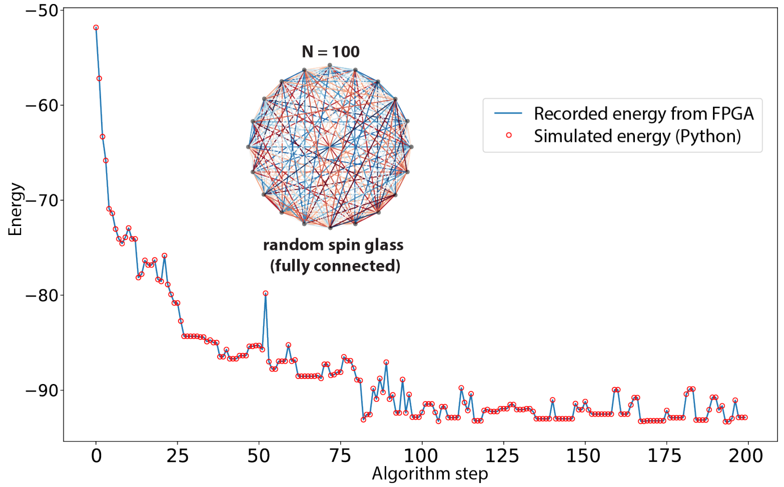

Supplementary Figure 16. **Comparison of FPGA and simulated (Python) outputs for a given random spin graph with** $N = 100$**.**

with rounded values. Several scaling factor and energy arrays are shown in Supplementary Figure **14**, in which we could see that scaling under 64 shows some deviation to the unrounded algorithm, and scaling above 1024 results in exactly the same sequence of state vectors.

▷ Correctness verification: we load the scaled test onto FPGA, and run 200 clock cycles and output state vector at each time step, with which we calculate the energy in Python. The FPGA-simulated energy and Python calculated energy is exactly the same, confirming the correctness of the implementation.

▷ Timing and complexity analysis: for this part we only output the state vector and clock count at the very end of 200 clock cycles. For our problem of size 100 by 100, a loop of 200 iterations cost 3803 cycles. On average each time step takes 19 clock cycles (the remaining 3 clock cycles are due to some overhead at the beginning of the run), which is in accordance with our analysis before.

The measured clock cycle per algorithm step is 19 cycles, which is exactly as expected: 19 = 10 + 8 + 1 in which 10 is the

cycles it takes to read the whole matrix (we are reading $r = 10$ rows at each clock cycle), 8 is the time for the binary addition tree, and an additional clock is required for noise injection and thresholding. In conclusion, we have implemented the PRIS for problem size $N = 100$ on an FPGA platform with a time per algorithm step of approximately 63 ns.

SUPPLEMENTARY REFERENCES

[1] R. M. Karp, "Reducibility among Combinatorial Problems," in *Complexity of Computer Computations*, pp. 85–103, Boston, MA: Springer US, 1972.

[2] F. Barahona, "On the computational complexity of Ising spin glass models," *Journal of Physics A: Mathematical and General*, vol. 15, pp. 3241–3253, oct 1982.

[3] F. Rendl, G. Rinaldi, and A. Wiegele, "Solving Max-Cut to optimality by intersecting semidefinite and polyhedral relaxations," *Mathematical Programming*, vol. 121, pp. 307–335, feb 2010.

[4] W. A. Little, "The existence of persistent states in the brain," *Mathematical Biosciences*, vol. 19, no. 1-2, pp. 101–120, 1974.

[5] J. J. Hopfield, "Neural networks and physical systems with emergent collective computational abilities.," *Proceedings of the National Academy of Sciences of the United States of America*, vol. 79, pp. 2554–8, apr 1982.

[6] J. J. Hopfield and D. W. Tank, "Neural computation of decisions in optimization problems," *Biological Cybernetics*, vol. 52, no. 3, pp. 141–152.

[7] C.-K. Looi, "Neural network methods in combinatorial optimization," *Computers & Operations Research*, vol. 19, pp. 191–208, apr 1992.

[8] P. Peretto, "Collective properties of neural networks: A statistical physics approach," *Biological Cybernetics*, vol. 50, pp. 51–62, feb 1984.

[9] D. J. Amit, H. Gutfreund, and H. Sompolinsky, "Spin-glass models of neural networks," *Physical Review A*, vol. 32, pp. 1007–1018, aug 1985.

[10] P. Horowitz and H. Winfield, "The Art of Electronics," *American Journal of Physics*, 1990.

[11] A. Azzalini and A. D. VALLE, "The multivariate skew-normal distribution," *Biometrika*, vol. 83, pp. 715–726, dec 1996.

[12] D. P. Landau and K. Binder, *A Guide to Monte Carlo Simulations in Statistical Physics*. 2009.

[13] Y. Shen, N. C. Harris, S. Skirlo, M. Prabhu, T. Baehr-Jones, M. Hochberg, X. Sun, S. Zhao, H. Larochelle, D. Englund, and M. Soljačić, "Deep learning with coherent nanophotonic circuits," *Nature Photonics*, vol. 11, pp. 441–446, jun 2017.

[14] L. Onsager, "Crystal Statistics. I. A Two-Dimensional Model with an Order-Disorder Transition," *Physical Review*, vol. 65, pp. 117–149, feb 1944.

[15] S. Friedli and Y. Velenik, *Statistical Mechanics of Lattice Systems*. Cambridge University Press, nov 2017.

[16] N. Metropolis, A. W. Rosenbluth, M. N. Rosenbluth, A. H. Teller, and E. Teller, "Equation of state calculations by fast computing machines," *The Journal of Chemical Physics*, 1953.

[17] W. K. Hastings, "Monte carlo sampling methods using Markov chains and their applications," *Biometrika*, 1970.

[18] N. C. Harris, G. R. Steinbrecher, M. Prabhu, Y. Lahini, J. Mower, D. Bunandar, C. Chen, F. N. C. Wong, T. Baehr-Jones, M. Hochberg, S. Lloyd, and D. Englund, "Quantum transport simulations in a programmable nanophotonic processor," *Nature Photonics*, vol. 11, pp. 447–452, jun 2017.

[19] N. C. Harris, Y. Ma, J. Mower, T. Baehr-Jones, D. Englund, M. Hochberg, and C. Galland, "Efficient, compact and low loss thermo-optic phase shifter in silicon," *Optics Express*, vol. 22, p. 10487, may 2014.

[20] P. L. McMahon, A. Marandi, Y. Haribara, R. Hamerly, C. Langrock, S. Tamate, T. Inagaki, H. Takesue, S. Utsunomiya, K. Aihara, R. L. Byer, M. M. Fejer, H. Mabuchi, and Y. Yamamoto, "A fully programmable 100-spin coherent Ising machine with all-to-all connections.," *Science (New York, N.Y.)*, vol. 354, pp. 614–617, nov 2016.

[21] R. Hamerly, T. Inagaki, P. L. McMahon, D. Venturelli, A. Marandi, T. Onodera, E. Ng, C. Langrock, K. Inaba, T. Honjo, K. Enbutsu, T. Umeki, R. Kasahara, S. Utsunomiya, S. Kako, K. I. Kawarabayashi, R. L. Byer, M. M. Fejer, H. Mabuchi, D. Englund, E. Rieffel, H. Takesue, and Y. Yamamoto, "Experimental investigation of performance differences between coherent Ising machines and a quantum annealer," *Science Advances*, 2019.

[22] R. Hamerly, L. Bernstein, A. Sludds, M. Soljačić, and D. Englund, "Large-Scale Optical Neural Networks Based on Photoelectric Multiplication," *Physical Review X*, vol. 9, nov 2019.

[23] N. H. Farhat, D. Psaltis, A. Prata, and E. Paek, "Optical implementation of the Hopfield model," *Applied Optics*, vol. 24, p. 1469, may 1985.

[24] X. Lin, Y. Rivenson, N. T. Yardimci, M. Veli, M. Jarrahi, and A. Ozcan, "All-Optical Machine Learning Using Diffractive Deep Neural Networks," apr 2018.

[25] D. Pierangeli, G. Marcucci, and C. Conti, "Large-Scale Photonic Ising Machine by Spatial Light Modulation," *Physical Review Letters*, 2019.

[26] S. Kirkpatrick, C. D. Gelatt, and M. P. Vecchi, "Optimization by simulated annealing.," *Science (New York, N.Y.)*, vol. 220, pp. 671–80, may 1983.

[27] Y. Nourani and B. Andresen, "A comparison of simulated annealing cooling strategies," *Journal of Physics A: Mathematical and General*, vol. 31, pp. 8373–8385, oct 1998.

[28] F. Glover and M. Laguna, "Tabu Search," in *Handbook of Combinatorial Optimization*, pp. 2093–2229, Boston, MA: Springer US, 1998.

[29] H. Cohn and M. Fielding, "Simulated Annealing: Searching for an Optimal Temperature Schedule," *SIAM Journal on Optimization*, vol. 9, pp. 779–802, jan 1999.
